# HQMSGAN: Enhancing Adversarial Robustness Using Hybrid Quantum-Classical Multi-Scale Generative Adversarial Network

**DOI:** 10.3390/e28101089

**Published:** 2026-10-05

**Authors:** Hamil Stanly, Mercy Shalinie Selvaraj, Riji Paul

**Affiliations:** Department of Computer Science and Engineering, Thiagarajar College of Engineering, Madurai 625015, Tamil Nadu, India; shalinie@tce.edu (M.S.S.); rijipaul@gmail.com (R.P.)

**Keywords:** quantum generative adversarial network (QGAN), adversarial robustness, multiscaling discriminator, adversarial training, white-box attack

## Abstract

The growing vulnerability of computer vision models to adversarial attacks has raised great concerns about the reliability of generative models in security-sensitive applications. Quantum generative models show strong potential for learning complex data distributions. However, the robustness of quantum generative models to adversarial perturbations and the effectiveness of adversarial training using quantum-classical generative architectures remain less explored. In this work, we evaluate quantum and classical generative network-inspired adversarial training to analyse whether quantum state-space representations can enhance adversarial robustness compared with their classical counterparts. We propose HQMSGAN, a novel hybrid quantum-classical generative adversarial network with multiscaling discriminators that denoise the adversarial attacks through adversarial training. During adversarial training, the proposed model performs quantum projections to learn rich nonlinear feature representations using quantum state encoding and performs feature coupling using quantum entanglement in the quantum state space. Thus, it learns the probability distribution of both the clean and adversarially perturbed data and tries to generate pure samples by reducing the loss over iterations in the quantum realm. The experiment is carried out by inducing four different adversarial attacks and defending them by adversarial training using the resources provided by AWS Braket’s quantum simulators in the Noisy Intermediate Scale Quantum (NISQ) era. The results provide favorable performance on metrics such as Structural Similarity Index Measure (SSIM), Peak Signal-to-Noise Ratio (PSNR), Fréchet Inception Distance (FID) score, Learned Perceptual Image Patch Similarity (LPIPS), and classification accuracy after performing adversarial training.

## 1. Introduction

The era of quantum AI has opened the door to numerous quantum-based generative AI and computer vision models that are extremely good at processing and classifying large volumes of image data using quantum properties. They excel in research data acquisition and analysis, accelerate data-driven decision-making, and automate repetitive tasks, thereby minimising human error in the detection and prediction of results. These high-tech Machine Learning (ML) models possess some intriguing properties that come under the study of Adversarial Machine Learning (AML) [1]. This was first identified in classical neural networks by Szegedy et al. [2]. Even highly potent deep learning models are susceptible to adversarial attacks. Adversarial samples are imperceptible noise deliberately calibrated as minute perturbations and applied to images to misclassify deep learning models in ways that go unnoticed by the human visual system [3,4]. As the machine learning models learn statistical patterns rather than structural patterns, a simple tweak is enough to carry out a significant attack. The study of AML in the quantum domain was first done by Lu et al. [5], where Quantum Machine Learning (QML) models became vulnerable to adversarial perturbations. Many research works have been done on various types of adversarial attacks in the classical domain when compared to the quantum domain. Research work on Quantum Adversarial Machine Learning (QAML) are limited in number and still in its infancy [6] in the NISQ era. Classical AML spans and affects several fields of study. For example, a successfully trained deep neural network in autonomous driving vehicles may predict a ’STOP’ traffic signboard as ’speed limit’ and vice versa if adversarial attacks influence them [7]. Similarly, a face recognition system may authorise unauthorised users in airports, banks or across a machine-critical application due to deliberately crafted adversarial samples [8,9]. It leads to illegitimate access control, impersonation, and a real-time threat to society. An adversarial attack in the medical field, when a malignant radiology image is mispredicted as normal, can be fatal and life-threatening [10,11]. The same is the case in agricultural fields when adversarial attacks affect the prediction of plant leaf diseases [12]. When normal plant leaves are misidentified as infected plant leaves, the agricultural sector allocates huge funds and resources to treat them, thus depleting huge computational resources [13]. These scenarios motivate us to focus on the analysis of adversarial attacks and to devise a defence technique whereby we can eliminate these attacks or at least reduce the impact of these intense attacks.

A generative AI model in computer vision, namely the Generative Adversarial Network (GAN) introduced by Ian Goodfellow [14], gained attention and popularity in 2014. This classical adversarial model can be employed to generate adversarial samples as well as defend against adversarial attacks [15]. The adversarial nature of GANs makes them notoriously unstable during training. They have evaluation metrics like the SSIM, PSNR ratio, FID score, and LPIPS, which are mathematical measures that quantify the probability distribution of the generated samples and show to what extent the probability distribution differs from the reference distribution. The classical GAN base version exhibits non-convergence, mode collapse, high computational cost, and training instability. Some of these issues can be overcome using a Wasserstein GAN-Gradient penalty (WGAN-GP) [16]. The WGAN model can handle mode collapse issues to some extent [17]. WGANs make use of the Earth Mover’s Wasserstein distance as a metric to measure the distance between two probability distributions. While WGANs make use of weight clipping, the WGAN-GP model imposes Lipschitz continuity instead of weight clipping. The gradient penalty aids in stabilising the training process. These classical generative models can be further advanced using quantum computation [18]. The complex patterns of images can be easily analysed using quantum bits that obey quantum principles such as superposition, entanglement, and interference. To counteract classical processing, QML offers quantum advantage in analysing and processing image samples more efficiently [19]. The advanced version of the classic GAN in the quantum realm is the Quantum Generative Adversarial Network (QGAN), which can be employed in quantum adversarial machine learning [5]. The adversarial nature in images can be learned using the hybrid QGAN models by employing adversarial training. A hybrid quantum-classical WGAN-GP architecture can perform adversarial training and act as a purification model to defend against adversarial attacks in the NISQ era [20]. QGANs are generative AI models with a quantum generation perspective that can be employed to defend against adversarial attacks [21]. Adversarial attacks may be launched in the form of single-step, multi-step, iterative or optimisation-based attacks [22]. For our research and analysis, we have taken four attacks, specifically the Fast Gradient Sign Method (FGSM), Basic Iterative Method (BIM), Momentum Iterative Method (MIM) and Projected Gradient Descent (PGD) attacks. Based on the adversarial knowledge, white-box attacks represent the strongest type of attack. They are white-box in nature, as the adversary takes control of the activation parameters, the architecture, and the entire dataset. Since the attacks are carried out across the gradient direction, these are classified as gradient-based attacks. These are serious threats that need attention and immediate intervention. Adversarial training is a machine learning defence technique that is highly resilient against adversarial attacks [23]. The aim is to supplement the training loop with clean and adversarial samples to learn the decision boundaries between the two sets of samples. The adversarial training technique tunes the model to be robust against adversarial attacks [24]. The primary goal of this research is to design a hybrid quantum-induced WGAN-GP model with multi-scaling discriminators that are trained adversarially using clean samples and adversarial samples to make it robust against adversarial attacks. The hybrid model is designed as a quantum-classical model to enhance adversarial robustness. The classical design extracts the primary features from the images. The quantum part employs the Parameterized Quantum Circuit (PQC) for efficient computation. It employs angle embedding using parameterized rotation gates to learn the different shades of the image and entanglement to perform feature coupling within the qubits and establish a spatial relationship among the pixels. Our major contributions are as follows:

- To develop a hybrid quantum defence framework, HQMSGAN, that employs a quantum-classical generator model along with classical multiscaling discriminators that can be adversarially trained to enhance robustness against adversarial attacks.
- To analyse the presence of white-box adversarial attacks in the form of a single-step FGSM attack, a multi-step iterative BIM attack, a momentum-based iterative MIM attack, and a multi-step optimisation-based PGD adversarial attack using the proposed model.
- To extensively evaluate the robustness of the proposed model using different quantum circuit configurations and measuring the related metric values by altering the qubit count and layer count.
- The model employs an adversarial training procedure to improve the adversarial robustness of the model using classical feature extraction using encoders and high-dimensional feature mapping and inter-feature coupling using the quantum module. The experimental results are evaluated using the confusion matrix, classification accuracy, SSIM, PSNR, FID score, and LPIPS metrics to understand the similarity of the image after the launch of the adversarial attack and to analyse its impact after the adoption of the proposed defence strategy.
- The experiments are carried out using State Vector 1 (SV1) quantum simulators built by AWS Braket, a novel computational platform in the NISQ era.

The structure of the paper is as follows: In Section 2, the background and preliminaries are presented. Section 3 discusses related work. In Section 4, the proposed methodology is elaborated. In Section 5, the experimental details are presented. The results and discussion, along with an ablation study, are elaborated in Section 6. In Section 7, the work is succinctly concluded, and future directions are discussed.

## 2. Background and Preliminaries

This section outlines the background and preliminaries of quantum computing and generative adversarial models.

### 2.1. Quantum Computing Preliminaries

This section explains the quantum basics, like quantum bits, quantum gates, and quantum circuits.

#### 2.1.1. Quantum Bits

The classical bit equivalent in quantum computing is the qubit, or quantum bit, represented by the notation |ψ〉. Qubits exist within the Bloch sphere in a superposition of |0〉 and |1〉 for encoding data in quantum computing as |ψ〉=α|0〉+β|1〉, where α and β are the complex probability amplitudes for measuring the states |0〉 and |1〉 respectively. The vector representation of |0〉 and |1〉 are represented as $|0\rangle =\left[\begin{array}{c} 1\\ 0 \end{array}\right]$ and, $|1\rangle =\left[\begin{array}{c} 0\\ 1 \end{array}\right]$ respectively. It should satisfy the condition ${\left|\alpha \right|}^{2}+{\left|\beta \right|}^{2}=1$.

#### 2.1.2. Quantum Gates

Single-qubit rotation gates $R_{x}$, $R_{y}$, $R_{z}$ are unitary transformations applied in the x, y, and z axes, respectively. The single-qubit unitary rotation in the *x* direction is given by $R_{x}\left(\theta \right)$. It is applied as shown in Equation (1):

(1) $$R_{x}\left(\theta \right)=\left[\begin{array}{cc} \cos\frac{\theta }{2} & -i\sin\frac{\theta }{2}\\ -i\sin\frac{\theta }{2} & \cos\frac{\theta }{2} \end{array}\right]$$

Similarly, the single-qubit unitary rotation in the *y* direction is given by $R_{y}\left(\theta \right)$. It is applied as shown in Equation (2):

(2) $$R_{y}\left(\theta \right)=\left[\begin{array}{cc} \cos\frac{\theta }{2} & -\sin\frac{\theta }{2}\\ \sin\frac{\theta }{2} & \cos\frac{\theta }{2} \end{array}\right]$$

and the single-qubit unitary rotation in the *z* direction is given by $R_{z}\left(\theta \right)$. It is applied as shown in Equation (3):

(3) $$R_{z}\left(\theta \right)=\left[\begin{array}{cc} e^{-i\theta /2} & 0\\ 0 & e^{i\theta /2} \end{array}\right]$$

Two-qubit transformations are applied by computing the Cartesian product of the two qubits. The CNOT gate performs entanglement between two qubits $|q_{0}q_{1}\rangle$, where $|q_{0}\rangle$ is the control qubit and $|q_{1}\rangle$ is the target qubit. If $|q_{0}\rangle$ is 0, then there is no change in $|q_{1}\rangle$, but if $|q_{0}\rangle$ is 1, then it flips the state of $|q_{1}\rangle$. Its matrix representation is as shown below:

(4) $$\mathrm{CNOT}=\left[\begin{array}{cccc} 1 & 0 & 0 & 0\\ 0 & 1 & 0 & 0\\ 0 & 0 & 0 & 1\\ 0 & 0 & 1 & 0 \end{array}\right]$$

The two-qubit representation can be extended to multi-qubit representations.

#### 2.1.3. Quantum Circuits

The quantum circuits are computational models with wires and quantum gates. The qubits are fed through the wires in which the gates are embedded to perform data encoding. The classical data preprocessing done on (x) yields the vector data ϕ(x). Unitary transformation $U_{enc}\left(x\right)$ on the qubit transforms the classical data ϕ(x) into its quantum counterpart, ψ(x) as shown below: The encoded quantum state ψ(x) is represented as $U_{\mathrm{enc}}{(\phi \left(x\right)|0\rangle }^{⊗n}.$ where ${|0\rangle }^{⊗n}$ represents the low-energy quantum state before the actual data encoding begins. The quantum circuits are often referred to as PQC where the parameters of the circuit are trainable. When the trainable parameters are optimised iteratively, the PQC becomes a Variational Quantum Circuit (VQC). The main components of the VQC are the single-qubit rotational gates that perform arbitrary rotations and the two-qubit CNOT gates that perform qubit entanglement. VQCs are employed in our work to design the quantum generator model using different configurations. The final section of the circuit is the measurement section. During measurement, the oscillating superpositional qubits of the quantum state converge to a definite classical bit.

### 2.2. Preliminaries of Generative Adversarial Model

Generative learning is employed to learn the presence of adversarial attacks and to purify the same using QGAN models. We adopt the structure of WGAN-GP, a traditional generative learning method and extend it to quantum processing to develop the proposed model, a quantum generative learning GAN model with three multiscaling discriminators.

#### 2.2.1. Classical WGAN-GP

The classical WGAN-GP comprises two models, namely the generator model *G* and the discriminator model *D*, and incorporates a gradient penalty to enforce the Lipschitz continuity constraint, thereby improving model performance and training stability [25]. The WGAN-GP model exhibits greater stability than the base GAN model by employing a gradient penalty, which ensures stable and smooth gradients for the generator, thereby mitigating convergence problems while defending against adversarial attacks.

#### 2.2.2. Hybrid Quantum-Classical GAN

The incorporation of a quantum component with classical WGAN-GP yields QGAN models that open new doors for computation in the quantum realm. QGAN models based on WGAN [26] are advanced generative AI computer vision models that can be employed to learn complex probabilistic data distributions using quantum properties to defend against adversarial attacks. A recent work by Tian et al. [27] proposed a hybrid quantum-classical WGAN-GP with an automated noise suppression mechanism. A simplified PQC circuit with reduced parameters and the quantum-inspired WGAN-GP assisted in smooth training convergence that helped to overcome the vanishing gradients. However, this model suffered from issues like the generation of blurry and vignetted images, along with occasional digit superposition. While experimenting with the F-MNIST dataset, blurriness and vignetting were reported due to the complex nature of the dataset. Occasional digit superposition was reported due to qubit constraints and limited training cycles in the NISQ era. These observations suggest the difficulty in preserving fine-grained details of images during image generation when incorporating quantum components. This work motivates us to focus on image representation while preserving the image semantics within an image purification pipeline.

The architecture of QGAN consists of two models, namely the generator model $G(\phi_{G})$, which is parameterized by a vector $\phi_{G}$, and the discriminator model $D(\theta_{D})$, which is parameterized by a vector $\theta_{D}$, engaging in combat with one another. The input to the generator is a noise vector represented by |Z〉. The discriminator model discriminates the output |Y〉 as either |true〉 or |false〉. The battle ends when the discriminator $\theta_{D}$ can no longer understand which is true and which is false. The QGAN optimisation is done as follows:

(5) $$\min_{\phi_{G}}\max_{\theta_{D}}\frac{1}{L}\sum_{\lambda =l}^{L}Pr\left(\left(D_{\theta D}\left(|\lambda \rangle ,\rho_{\lambda }^{R}\right)=|true\rangle \right)\;\cap \left(D_{\theta D}\left(|\lambda \rangle ,\rho_{\lambda }^{G}\left(z\right)\right)=|false\rangle \right)\right)$$

The training of the quantum GAN yields the Nash equilibrium between the generator and discriminator:

(6) $$\begin{array}{c} \max_{\phi_{G}}L_{G}\left(\phi_{G},\theta D\right)\\ \max_{\theta_{D}}L_{D}\left(\phi_{G},\theta D\right) \end{array}$$

## 3. Related Work

This section outlines the pertinent research and analysis on adversarial attacks, various adversarial defence techniques, and the use of generative AI models to defend against them.

### 3.1. Adversarial Attacks

Adversarial attacks are significantly impactful and are crafted stealthily with adversarial knowledge. If the complete knowledge of the internal model is known, it comes under the category of a white-box attack. In the case of a black-box attack, this valuable information is concealed. A potent white-box attack can be launched when all model parameters, activations, gradients, and training datasets are accessible. The study on white-box adversarial attacks dates back to 2014, when Szegedy et al. [2] introduced it. The white-box attack is the strongest type of adversarial attack that can be launched. So we focus our research and analysis on the following four types of white-box attacks.

#### 3.1.1. FGSM Adversarial Attack

Fast Gradient Sign Method (FGSM) is a one-shot adversarial attack launched in the direction of the gradients of the loss function [28]. The aim is to maximise the loss while preserving the visual aesthetics of the image. A minute, well-defined perturbation is added intentionally along the gradients of the image to create a pixel-wide modification to the image in the direction specified by the adversary. The perturbations are so mild that they go unnoticed by the human visual system. It is a white-box attack, as there is full access to the architecture and the model parameters θ. This allows for an easy computation of the gradients of the image. The minute adversarial perturbation η is devised as shown below:

(7) $$\eta =\epsilon \cdot \mathrm{sign}\left(\nabla_{x}J\left(\theta ,x,y\right)\right)\;$$

where the multiplier constant ϵ ensures that the perturbations applied to the image (x,y) are extremely minimal. The output label is *y* for the corresponding *x*. The gradient loss function, J(θ,x,y), is maximised to increase the level of misclassification. The sign (·) is the sign function, and $\nabla_{x}\left(\cdot \right)$ is the gradient of the input image. The perturbation is added to the clean image *x* to generate the adversarial sample $x^{\prime }$ as shown below:

(8) $$x^{\prime }=x+\eta$$

The adversarial image $x^{\prime }$ can be generated by just a single-step adversarial perturbation η. It is a simple and fast attack that makes use of a one-time calculation to carry out the attack.

#### 3.1.2. BIM Adversarial Attack

BIM, also known as iterative FGSM (iFGSM), is an extension of the FGSM attack [29]. It is a gradient-based white-box adversarial attack initiated using an iterative process with multiple steps instead of a single-step FGSM attack. The adversarial attack is carried out as follows:

(9) $$\begin{array}{c} x_{0}=x\\ x_{t+1}={\mathrm{Clip}}_{x,\epsilon }\left(x_{t}+\alpha \cdot \mathrm{sign}\left(\nabla_{x}J\left(x_{t},y\right)\right)\right) \end{array}$$

#### 3.1.3. MIM Adversarial Attack

MIM attack is an extension of the BIM attack. It is initiated by accumulating the momentum term to perform an optimisation-based update [30]. By applying momentum, it tries to overcome the local maxima issues. This attack is stronger than the BIM attack. The gradient of the loss function is computed as follows:

(10) $$\;g_{t}=\nabla_{x}F\left(\theta \left(x_{t}^{adv}\right),y\right)\;$$

The gradients are normalized using the $L_{1}$ norm regularization. The gradient is updated by adding the current gradient to the accumulated momentum as shown below.

(11) $$\begin{array}{cc} {\hat{g}}_{t} & =\frac{g_{t}}{{∥g_{t}∥}_{1}}\\ g_{t+1} & =\mu g_{t}+{\hat{g}}_{t}\; \end{array}$$

where μ is the momentum factor or decay factor multiplied by the current gradient value.

(12) $$x_{t+1}^{adv}={\mathrm{Clip}}_{x,\epsilon }\left(x_{t}^{adv}+\alpha \mathrm{sign}\left(g_{t+1}\right)\right)\;$$

#### 3.1.4. PGD Adversarial Attack

**Figure 1:**
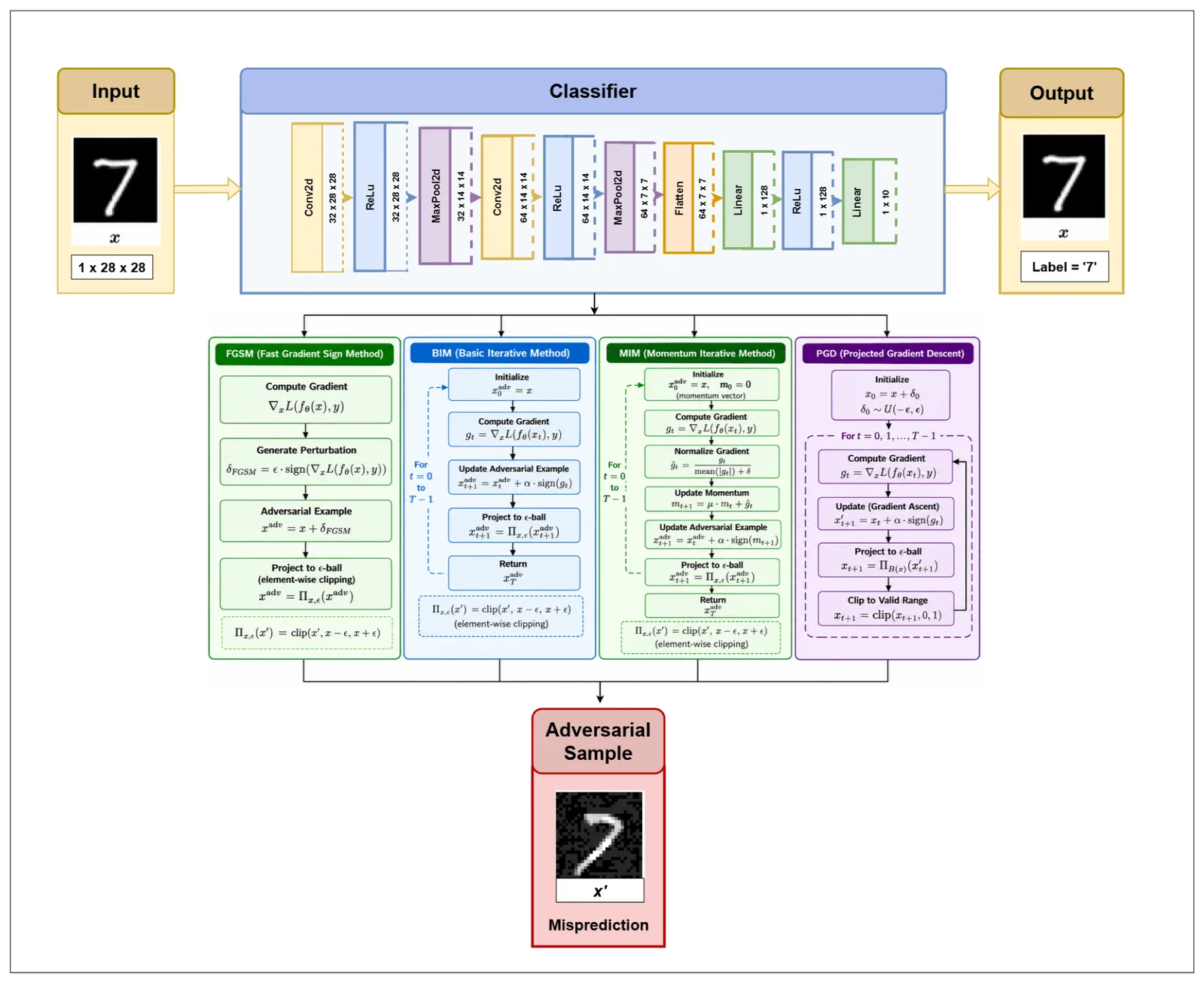
Adversarial attack generation using the FGSM, BIM, MIM and PGD algorithms.

PGD is a prevalent form of multi-step adversarial attack that can be easily launched without much time and effort. This iterative attack was first launched by Madry et al. [31]. It is the strongest form of white-box attack. The one-shot FGSM attack is iterated repeatedly to form the PGD attack technique. It gets stronger in generating adversarial samples after each iteration. The adversarial sample at t+1 iteration, $x_{t+1}$ is computed by using the $x_{t}$ adversarial image at iteration *t* as shown below:

(13) $$\begin{array}{c} x_{0}=x+\delta \\ x_{t+1}=\Pi_{\epsilon }\left(x_{t}+\alpha \cdot \mathrm{sign}\left(\nabla_{x}J\left(\theta ,x,y\right)\right)\right)\; \end{array}$$

where $\Pi_{\epsilon }$ represents the projection operator that keeps the perturbation within the level specified by ϵ. The step size for each iteration is specified by α. It generates an adversarial image with maximum loss and minimum perturbation. The architecture of Convolutional Neural Network (CNN) used as a classifier for the input samples is shown in Figure 1. The classifier model predicts clean samples as pure and misclassifies attack samples as adversarial when attacks such as FGSM, BIM, MIM, and PGD are applied to them.

### 3.2. Adversarial Defence

Many adversarial defence techniques are being proposed to defend against adversarial attacks. Adversarial defence techniques that can fully comprehend adversarial attack strategies are still under research and analysis. The detailed related work is listed in Table 1. This table bridges the gap in current analysis and extends adversarial defence into a new dimension by addressing the limitations found in existing literature.

#### 3.2.1. Adversarial Robustness Stratergies

Simple image reconstruction techniques can repair the perturbations contained in the image to some extent [41]. The feature squeezing, feature denoising, and image smoothing techniques may often remove the vital details from the image while making the model robust. Sophisticated neural network models can defend against adversarial attacks with architectural tweaks. The MagNet [33] employs an autoencoder that comprises a detector network and a reformer network for defending against adversarial attacks. The detector network differentiates between the clean and adversarial samples, and the reformer network reforms the adversarial samples and moves them close to clean samples. The paper discusses the need for a more powerful detector and reformer to defend against powerful attacks. This shows us that architectural refinement is necessary to combat adversarial attacks. Another main drawback is that the classification accuracy for CW attacks is 0% for specific conditions, which is not acceptable. The methods discussed till now made use of neural networks to combat adversarial attacks. There are GAN-based architectures that can act as a defence framework. The Defence-GAN [34] is a GAN-based mechanism introduced to defend against adversarial attacks. The model used is basically a WGAN model with gradient descent iterations, random restarts, and a classifier to defend against adversarial attacks. The reconstruction error is the metric used to compare the legitimate images and perturbed images. They fully rely on the training stability and the generative power of WGAN for performing the defence. Tuning the sensitive gradient descent parameters may be challenging. AdvGAN [42] makes use of a GAN-based method with a feed-forward generator model to generate high-perceptual-quality adversarial samples that closely resemble legitimate samples. They face issues like complex model training, which drain time and effort. The defence against adversarial attacks is done by using a denoising technique that projects the images within the range of the generator model. Some research works focused on purification methods. AdvPurify-GAN [43] makes use of a GAN model to perform adversarial purification on medical images. They remove the adversarial noise from the image and purify it by employing a GAN model with stabilised training. The major issues are that they are vulnerable to adaptive attacks and their defence strategy is highly dependent on the choice of the hyperparameters. They also face generalisation challenges while dealing with two- and three-dimensional images. PuriFlow [44] employs a combined strategy using a super-resolution technique and a diffusion technique to combat adversarial attacks. They perform upsampling followed by downsampling on the attack image to reduce the feature discrepancies and to enhance the visual fidelity of the image by reducing the distortion of textures. If the super-resolution technique is performed solely without the diffusion step, then the Mean Squared Error (MSE) increases, leading to model inversion. Wang et al. [45] have proposed an iterative window mean filter to perform robust adversarial purification using denoising diffusion models. The adversarial robustness is increased by rendering strong suppression of perturbations. This eradicates the potent features from the images, causing blurring effects, thereby impacting low classification accuracy. Wang et al. [46] have employed noise injection and multilayer filters to defend against adversarial attacks. Excessive filtering results in robustness–generalisation trade-offs and may slowly eliminate the primary image features.

#### 3.2.2. Adversarial Training Strategies

Adversarial training is one of the most promising defence strategies to defend against adversarial attacks [23]. Much work has been done on adversarial training. Tramer et al. [32] have proposed ensemble adversarial training. In this approach, the perturbations are added from other static, pre-trained models. This process improves the diversity of perturbations, but slows down the convergence speed. The convergence rate of this technique is slower than traditional adversarial training. They exhibited low performance in certain settings. GAN-based models are efficient in adversarial training [47]. Laykaviriyakul et al. [36] have proposed a Collaborative defence GAN to reconstruct the legitimate samples from the adversarial samples using two generators and two discriminators for forward and reverse mapping. The strategy used for defence is noise filtration and adversarial training. The procedure for noise filtration is not clearly elaborated. They provide defence against both white-box and black-box adversarial attacks. It incurs an additional cost that involves reconstructing the adversarial images. They employ a more complex U-Net deep learning model for defending against attacks. The complex models slow down the reconstruction process. Also, for some datasets, the performance of the model degrades drastically.

The robust training proposed by Baytas et al. [37] protects the classical deep learning models from adversarial attacks. The lightweight generator model of GAN generates perturbations from a random vector in a single step, and it does not require any gradient information. They have listed some limitations. That is, while defending against the PGD attack, the robustness performance drops below the state-of-the-art technique. Certain datasets incurred increased training time. Zhao et al. [38] have proposed a multi-guided adversarial training technique. It is a weighted ensemble training method to defend against adversarial attacks. The two guiding strategies, namely self-guidance and expert guidance, assist in marking the decision boundaries between the clean and adversarial classes. At times, they face difficulty in balancing decision boundaries. The difficulty of balancing decision boundaries. They face overfitting issues if the guidance components are not perfectly balanced. Some adversarial training was done in the quantum domain. Kehoe et al. [35] proposed a Quantum Restricted Boltzmann Machine (QRBM), a generative probabilistic graphical model that processed MNIST datasets of two dimensions, namely, 28×28 greyscale images and rescaled 7×7 binary images for defending against adversarial attacks. They used quantum annealers for quantum-enhanced sampling. They have experimented using only the MNIST dataset. The authors admit that they do not have any strong claim with regard to quantum advantage. The experiment section does not specify the quantum bits or other quantum configurations adopted for their experimentation. Shi et al. [40] have proposed a hybrid Quantum Convolutional Neural Network (QCNN) architecture to defend against white-box adversarial attacks. They have asserted that they have done a comparison with four classical models. But the comparison is not highlighted. Also, the values tabulated in the tables show the robustness comparison of models and the adversarial robustness comparison of datasets. However, the quantum configuration for which these values were tabulated is not specified. They have used nine configurations of quantum circuits for experimentation. We have adopted a similar configuration for our experimentation and comparison.

#### 3.2.3. Multiscaling Stratergies

Several classical methods employ multiscaling techniques to enhance the learning strategy. Rao et al. [48] proposed a Multi-scale GAN (MS-GAN) utilising parallel class activation maps for image reconstruction. Lee et al. [49] augment the discriminative efficacy of the discriminator through the utilisation of multi-scale representations. Ma et al. [50] propose a saliency-discriminated GAN with paired discriminators to evaluate the quality of salient and nonsalient regions. Karnewar et al. [51] proposed the multi-scale gradient GAN, an efficient technique that utilises gradient flow from the discriminator to the generator across multiple scales, thereby enhancing stability in image synthesis. Shao et al. [52] utilised a conventional WGAN with multi-scale discriminators. A low-intensity adversarial attack is launched, and the framework employing a multi-scale GAN successfully mitigates it. The multi-scale information facilitates effective denoising and enhances defence capability. The disadvantages of this methodology include prolonged training duration and insufficient feature-level analysis. We employ this idea of multi-scale discriminators in our research to achieve better QGAN stability, learning ability and robustness to defend against adversarial attacks. Related studies indicate a substantial research gap in exploring robust quantum methodologies for defending against adversarial attacks. Our proposed method has undergone extensive analysis by employing different quantum circuit configurations. Adversarial training employing multiscaling discriminators is more advantageous in terms of GAN stability and reducing the impact of adversarial attacks. Based on the literature survey, we adopt classification accuracy metrics to measure the model’s performance on clean data and to study the effect of adversarial attacks after the dataset is contaminated. We evaluate the quality of the adversarial attack samples and defence samples using four metrics, namely the SSIM, PSNR ratio, FID score, and LPIPS. Thus, we propose the HQMSGAN model, which utilises multiscaling discriminators to enhance the training stability of the quantum generator model and to improve the image quality of the purified samples.

## 4. Proposed Methodology

**Figure 2:**
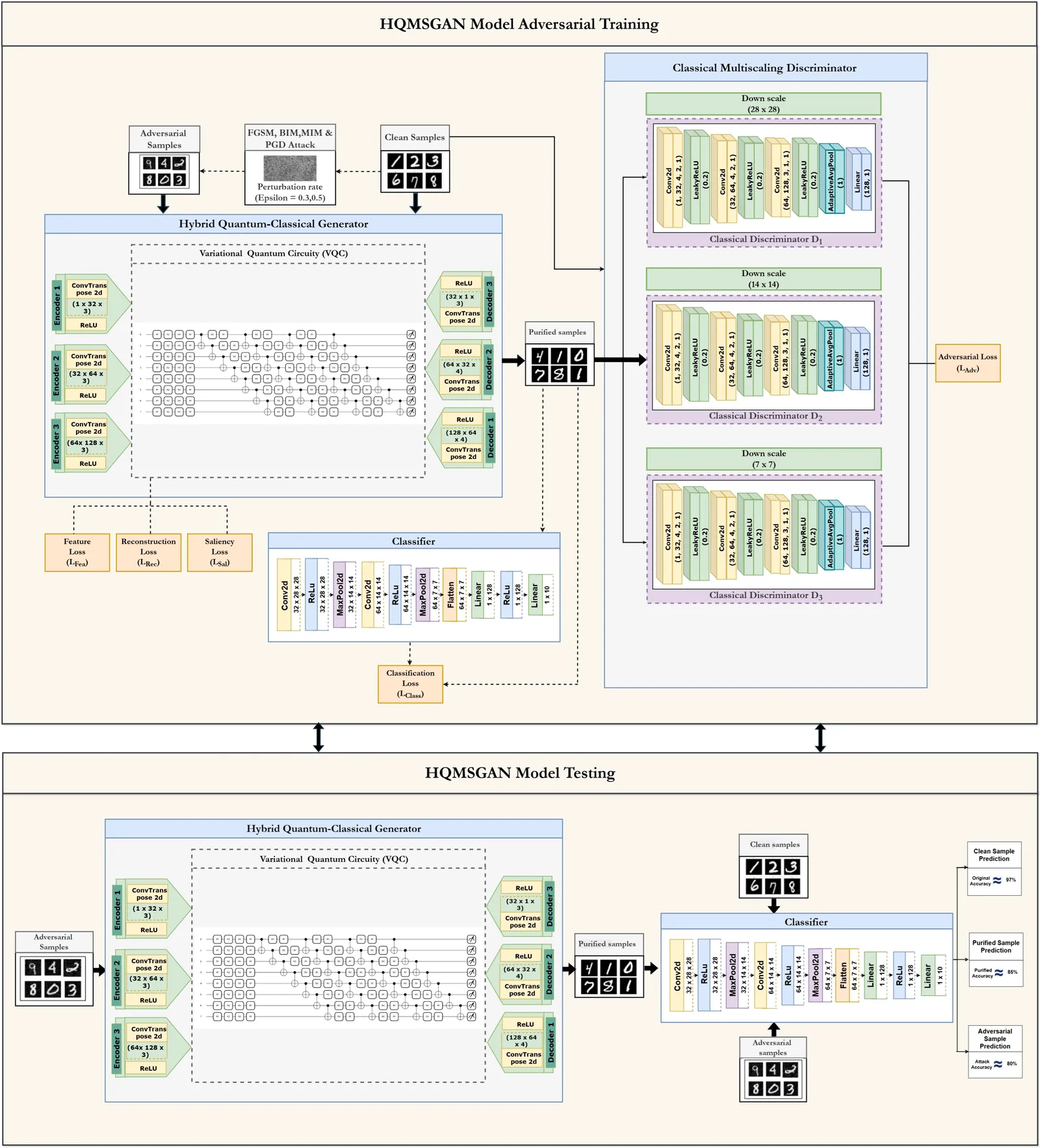
Block diagram of HQMSGAN: Model training and testing. Model training: The hybrid quantum-classical generator model with the multiscaling discriminators performs adversarial training. Model testing: Testing is carried out using the classifier model that predicts the output.

**Figure 3:**
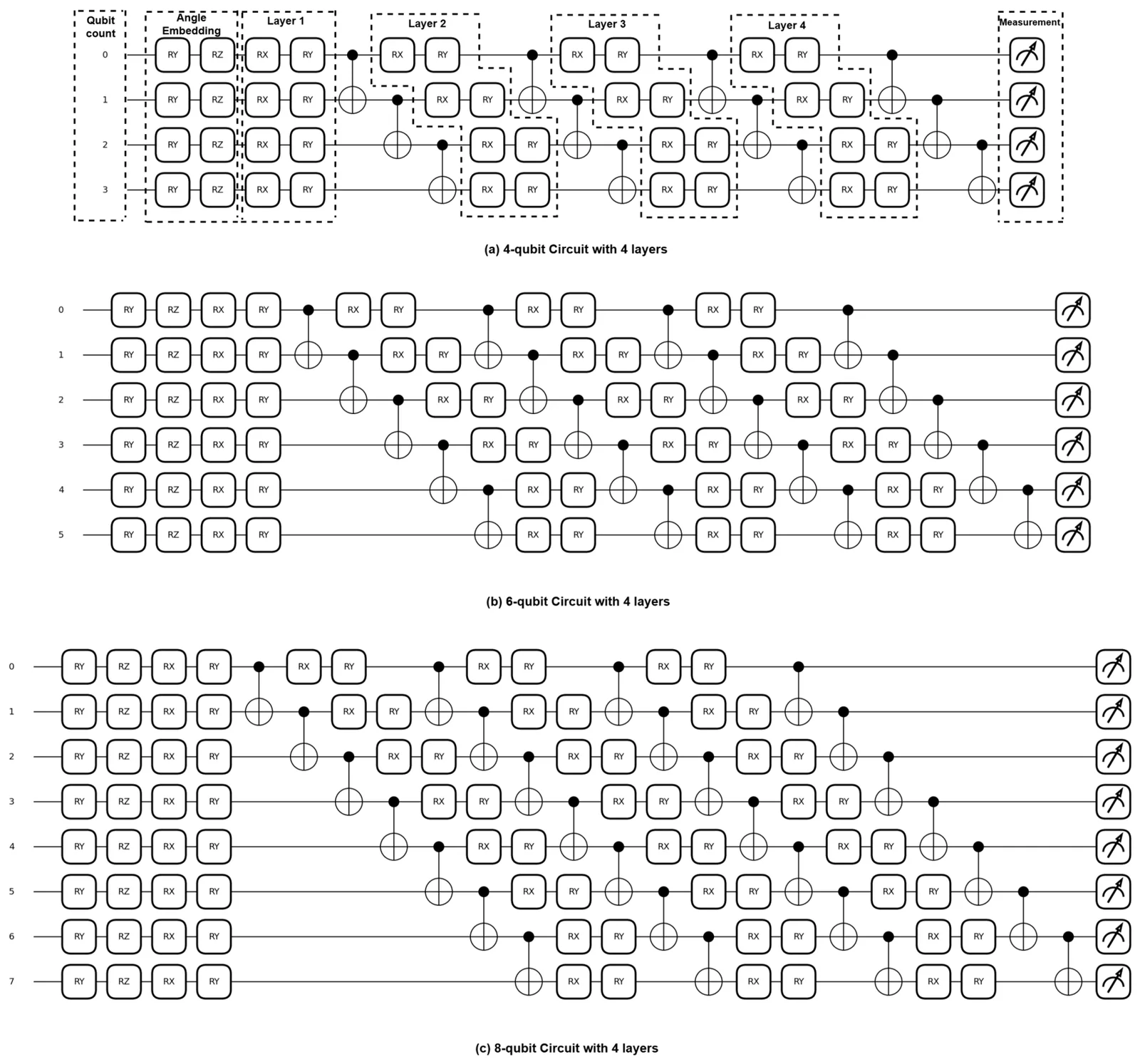
Variational Quantum Circuit: A total of 9 different VQC configurations are employed for experimentation. Among them, only three different configurations are displayed: (**a**) 4-qubit circuit with 4 layers ($q_{4}l_{4}$), (**b**) 6-qubit circuit with 4 layers ($q_{6}l_{4}$), (**c**) 8-qubit circuit with 4 layers ($q_{8}l_{4}$).

A hybrid quantum-classical generator with multiscaling discriminators that perform adversarial training is proposed to defend against adversarial attacks in the NISQ era. Adversarial training is a two-step optimisation problem solved by the hybrid quantum-classical GAN. It performs a min-max optimisation by finding the maximum perturbation ϵ while minimising the loss. It can be formulated as shown below:

$$\min_{\theta }\;E_{\left(x,y\right)∼P_{data}}\left[\max_{∥\epsilon ∥\leq \epsilon_{\max}}L\left(f(x+\epsilon ;\theta ),\;y\right)\right]$$

where (x,y) are the sample images within the data distribution $P_{data}$. *y* is the label corresponding to the image *x*. f(x;θ) represents the network parameter θ, ϵ is the induced perturbation, and $\epsilon_{max}$ is the maximum induced perturbation. L(·) is the cross-entropy loss function. With much fewer parameters, the quantum-classical GAN learns to generate pure samples from a mix of clean and adversarial samples. The block diagram of the proposed HQMSGAN model, as shown in Figure 2, comprises the adversarial training phase and the testing phase. The training phase consists of a hybrid quantum-classical model with three discriminators, each downscaled to a different scale. The adversarial training phase takes the clean samples and adversarial samples as input to learn the difference between the probability distributions of both sets of samples. It is an intensive purification process involving hybrid quantum and classical models. The quantum-classical generator model is an integration of encoders, quantum circuits, and decoders for enhancing adversarial robustness against adversarial attacks. The encoders extract features and learn the most crucial features from input samples. It preserves the spatial size of the images while extracting the visual features and textures of the images. The 28-by-28 image input is fed to three encoders with 3×3 kernels utilizing 1 to 32, 32 to 64, and 64 to 128 channels. Strided convolutions are applied to the first, second, and third convolutional layers with a stride value of 1, 2, and 2, respectively, enhancing spatial downsampling. The ReLU activation function is applied to retain the positive values and to block the negative values. The input image of dimension 1×28×28 is transformed to 32×28×28 and then to 64×14×14 and finally results in a 128×7×7 dimension. Thus, the encoder compresses the most critical high-dimensional classical data into low-dimensional data that can be fed to a VQC. These flattened latent representations are supplied as inputs to the quantum configurational circuits of 4, 6, and 8 qubits, with each feature mapped to a qubit. A total of 9 quantum circuit configurations are used: 4-qubit, 6-qubit, and 8-qubit circuits with 4, 6, and 8 layers for research and analysis. Among the nine VQC designs, three VQCs are shown in Figure 3. They are a 4-qubit circuit with 4 layers ($q_{4}l_{4}$), a 6-qubit circuit with 4 layers ($q_{6}l_{4}$), and an 8-qubit circuit with 4 layers ($q_{8}l_{4}$). As shown in the figure, 4 qubits are fed as input to the angle-embedding layer. The angle-embedding layer encodes the classical input and maps it to quantum data by performing rotations in the $R_{Y}\left(\theta_{1}\right)$ and $R_{Z}\left(\theta_{2}\right)$ directions as specified by the angles $\theta_{1}$ and $\theta_{2}$. During angle embedding, the initial state is transformed from the initial feature vector $\vec{x}$ to single-qubit rotations by changing the probability amplitudes of the qubits. The initial feature vector contains a list of features $\left[x_{1},x_{2}\ldots x_{n}\right]$. Angle encoding transforms the data from a classical state to a quantum state. It is done by $|\Psi_{encode}\left(\vec{x}\right)\rangle ={⨂}_{i=1}^{n}R_{Y}\left(\theta_{i}\right)|0\rangle$ $=\cos\left(\frac{\theta_{i}}{2}\right)|0\rangle +\sin\left(\frac{\theta_{i}}{2}\right)|1\rangle$ The single-qubit rotation gates $R_{x}\left(\theta_{1}\right),R_{y}\left(\theta_{2}\right),R_{z}\left(\theta_{3}\right)$ rotate within the Bloch sphere by the angle specified by $\theta_{1},\theta_{2}\;\mathrm{and}\;\theta_{3}$. These angles are the trainable weights. The two-qubit CNOT gate is shown as a solid black dot (•) from the control qubit to the target qubit, shown by a crossed target circle ⊕. Following angle embedding, the qubits pass through a layer of rotation gates. The rotation gates are crucial in adjusting the input noise vector when the preprocessed data is fed into the PQC’s embedding layer. It then moves through the entanglement layer, which makes use of the CX gates. The circuit wires are entangled with each other to improve its inter-feature coupling capacity. The quantum states are linked to one another when the qubits in the circuit are entangled. The generator executes an embedding to transform classical states into the quantum Hilbert space. The VQC performs latent adversarial feature refinement before performing image reconstruction. The quantum state is measured with Pauli-Z gates that compute expectation values of VQC observables, resulting in 4, 6, and 8 quantum features. The measurement results pass through the projection layer, which in turn is fed as input to subsequent decoders. The quantum gradients are calculated using the parameter-shift rule. The transposed convolutional layers in the decoders reconstruct the images from latent space representations. It performs latent upscaling and reconstruction of data to preserve the structural fidelity of the image. Over many iterations, the generator performs residual correction by learning the differences between the distribution of clean and adversarial samples. Learning this difference leads to the generation of purified samples by narrowing the boundary between their respective probability distributions. During learning and optimisation, the generator model of HQMSGAN experiences five losses, namely the adversarial loss, reconstruction loss, classification loss, feature loss and the saliency loss. The generator losses are computed as follows: The loss incurred by the generator while learning to generate pure samples during the adversarial game is measured as the adversarial loss.

(14) $$G_{Adv_{Loss}}=-E_{\tilde{x}∼P_{g}}\left[D(\tilde{x})\right]$$

where $D(\tilde{x})$ denotes the critic score for the purified image. To keep the purified image close to the clean image at the pixel level, the reconstruction loss consisting of MSE and L1 is computed as follows:

(15) $$G_{Rec_{Loss}}=0.5L_{MSE}+0.5L_{L1}$$

where MSE is the Mean Squared Error and L1 is the Mean Absolute Error, which is calculated as $L_{MSE}=MSE\left(I_{puresample},I_{cleansample}\right)$ and $L_{L1}=L1\left(I_{puresample},I_{cleansample}\right)$. The classification loss is the cross-entropy measure incurred while classifying the purified samples to the ground truth labels.

(16) $$G_{Class_{Loss}}=CE\left(C\left(I_{puresample}\right),y\right)$$

where CE is the cross-entropy loss.

The feature loss is the MSE between the feature representations of clean and purified images. The feature representation is learned by the classifier by extracting the intermediate features of both the clean and purified images with the classifier.

(17) $$G_{Fea_{Loss}}=MSE{∥\phi \left(I_{puresample}\right),\;\phi \left(I_{cleansample}\right)∥}_{2}^{2}$$

where ϕ(·) represents the features of the images. After purification of image samples, the most important visual regions of the image are preserved. The loss incurred during the preservation of vital regions is measured as saliency loss. The gradient-based saliency maps are computed for clean and purified images to preserve the salient regions of the clean image. The saliency map is obtained from the absolute maximum of the gradient classifier output on the clean and purified image. It is measured as the $L_{1}$ distance between the clean and purified samples.

(18) $$G_{\mathrm{SalLoss}}={∥S_{puresample}-S_{cleansample}∥}_{2}^{2}$$

The modified generator loss is implemented as the weighted summation of the above losses as shown in Equation (19).

(19) $$G_{Loss}=0.001G_{Adv_{Loss}}+15G_{Rec_{Loss}}+8G_{Class_{Loss}}+15G_{Fea_{Loss}}+10G_{Sal_{Loss}}$$

The weight coefficients were chosen as hyperparameters according to the different objective functions of each loss. The weight coefficients were not assumed to be the same value, but were chosen according to numerical scales and optimization roles. The reconstruction loss, classification loss, feature loss, and the saliency loss correspond to image fidelity and semantic preservation of the image. Hence, larger coefficient constraints are added to it, whereas the adversarial loss provides a distribution-level constraint. So smaller coefficient constraints are assigned to it. These constraints were kept unaltered throughout the experiments. To assess the individual contribution of each loss component, an ablation study was performed. The generator loss exhibits some oscillations, but the bounded behavior of the loss indicates stable adversarial training.

The multiscaling discriminator model functions as a critic and has a classical design. An in-depth exploration of discriminators led to the development of a multi-scale generative–discriminative learning design. A multiscale discriminator operates across multiple scales to enhance fine-grained, pixel-level details in the image and to remove visual artefacts. It aids in stability during image synthesis and facilitates image reconstruction during purification. Competing with the quantum generator model are three classical multiscaling discriminator models: $D_{1}$, with a scaling factor of 28×28, $D_{2}$, with a scaling factor of 14×14, and $D_{3}$, with a scaling factor of 7×7. Learning at different scales is a hierarchical approach that aids in learning the global and local features of clean and adversarial samples. When it employs the three-level scaling strategy to analyse complex data distributions, it learns better with different resolution scales. It learns during forward and backward propagation, thus reducing the risk of mode collapse, a major issue while employing GANs. Each of the three discriminators has the same internal structure. Each discriminator is a deep neural network comprising convolutional layers, leaky ReLU layers, instance normalisation, and a linear layer. The convolutional layer extracts local features and learns spatial hierarchies. We utilise a leaky ReLU layer instead of ReLU to mitigate the dying ReLU issue. It maintains a minimal gradient even with negative inputs, facilitating gradient flow and enhancing training stability. Instead of batch normalisation, instance normalisation is employed to normalise the feature map for each instance. The primary aim of instance normalisation is to eliminate contrast variations among images. After passing through various layers that extract the intricate features from the image, the output is measured from the fully connected layer instead of measuring from the sigmoid layer. The fully connected layer converts the features of the image into a Wasserstein critic score, resulting in an unbounded scalar value. The multi-scaling method identifies the significant features of the clean samples that can be incorporated into the latent vector to produce adversarial samples. This thereby reduces the distance between the original sample and the adversarial sample, thereby enhancing the QGAN’s generating capacity. The gradient penalty in WGAN-GP helps alleviate convergence problems during adversarial training. The downscaling technique enhances training stability. The adversarial attack algorithm and the proposed defence algorithm are shown in Algorithm 1 and Algorithm 2, respectively. Our defence algorithm’s primary mathematical goal is to minimise the distance between the attack samples and purified samples. After several refinements and iterations of adversarial training, the hybrid model generates purified samples that are close to the real samples. The generator learning rate $lr_{g}$ and the discriminator learning rate $lr_{d}$ play an important role in generating pure samples. A fine-tuned learning rate fixes the GAN instability issues pointed out as a major disadvantage in many research articles. The loss experienced by the multi-scale discriminator is shown below:

(20) $$D_{Loss}=E_{\tilde{x}∼P_{g}}\left[D(\tilde{x})\right]-E_{x∼P_{r}}\left[D(x)\right]+\lambda \;*GP$$

where λ is the coefficient of gradient penalty. The gradient penalty (GP) used in WGAN-GP is as follows:

(21) $$GP=E_{\hat{x}∼P_{\hat{x}}}\left[{\left({∥\nabla_{\hat{x}}D\left(\hat{x}\right)∥}_{2}-1\right)}^{2}\right]$$

where $\hat{x}∼P_{\hat{x}}$ are the generated samples between real and fake samples, $\nabla_{\hat{x}}D\left(\hat{x}\right)$, is the discriminator’s output and ${∥\cdot ∥}_{2}$ is the $L_{2}$ norm.

(22) $$D_{loss_{1}}=E_{\tilde{x}∼P_{g}}\left[D_{1}\left(\tilde{x}\right)\right]-E_{x∼P_{r}}\left[D_{1}\left(x\right)\right]+\lambda_{1}\;*GP$$

(23) $$D_{loss_{2}}=E_{\tilde{x}∼P_{g}}\left[D_{2}\left(\tilde{x}\right)\right]-E_{x∼P_{r}}\left[D_{2}\left(x\right)\right]+\lambda_{2}\;*GP$$

(24) $$D_{loss_{3}}=E_{\tilde{x}∼P_{g}}\left[D_{3}\left(\tilde{x}\right)\right]-E_{x∼P_{r}}\left[D_{3}\left(x\right)\right]+\lambda_{3}\;*GP$$

The modified discriminator loss is the summation of all the losses of the multiscaling discriminators. It is shown in Equation (25).

(25) $$\;D_{loss}=D_{loss_{1}}+D_{loss_{2}}+D_{loss_{3}}$$

$\lambda_{1},\lambda_{2},\lambda_{3}$ are the coefficients of the gradient penalty that regulate the loss to achieve optimised performance.
**Algorithm 1** Adversarial Attack: Attack generation algorithm**Require:** Trained Classifier $N_{\theta }$, Clean sample ${\left(x,y\right)}_{clean}$, perturbation rate: ϵ **Ensure:** Adversarial sample ${\left(x_{j},y_{j}\right)}_{Adv}$, where $x_{j}\in X$ is the adversarial image, $y_{j}\in Y$ is    the label corresponding to the image
  1:Method: FGSM adversarial attack  2:Compute the gradient: $g_{t}\leftarrow \left(\nabla_{x}J\left(\theta ,x,y\right)\right)$  3:Select the perturbation: $\eta =\epsilon \cdot \mathrm{sign}\left(g_{t})\right)$  4:Initiate FGSM attack: $x_{fgsm}\leftarrow {\left(x,y\right)}_{Clean}+\eta$  5:${\left(x,y\right)}_{Adv}\leftarrow x_{fgsm}$  6:**return** ${\left(x,y\right)}_{Adv}$  7:Method: BIM adversarial attack  8:Initialise: $x_{0}^{adv}\leftarrow x$  9:**for** t=0 to t=(T−1) **do**10:      Compute the gradient: $g_{t}\leftarrow \nabla_{x}J\left(\theta \left(x_{t}^{adv}\right),y\right)$11:      Initiate BIM attack $x_{t+1}^{adv}\leftarrow x_{t}^{adv}+\alpha \mathrm{sign}\left(g_{t}\right)$12:      Clip ϵ within the range $x_{bim}:x_{t+1}^{adv}\leftarrow {\mathrm{Clip}}_{x,\epsilon }\left(x_{t+1}^{adv}\right)$13:      ${\left(x,y\right)}_{Adv}\leftarrow x_{bim}$14:**end for**15:**return** ${\left(x,y\right)}_{adv}$16:Method: MIM adversarial attack17:momentum factor μ18:Initialise: $x_{0}^{adv}\leftarrow x$19:Initialise momentum term: $g_{0}\leftarrow 0$20:**for** t=0 to t=(T−1) **do**21:      Compute the gradient: $g_{t}\leftarrow \nabla_{x}F\left(\theta \left(x_{t}^{adv}\right),y\right)$22:      Normalise the gradients using $L_{1}$ norm ${\hat{g}}_{t}\leftarrow \frac{g_{t}}{{∥g_{t}∥}_{1}}$23:      Add the momentum term: $g_{t+1}\leftarrow \mu g_{t}+{\hat{g}}_{t}$24:      Initiate MIM attack: $x_{t+1}^{adv}\leftarrow x_{t}^{adv}+\alpha \mathrm{sign}\left(g_{t+1}\right)$25:      Clip ϵ with minimum ϵ: $x_{mim}:x_{t+1}^{adv}\leftarrow {\mathrm{Clip}}_{x,\epsilon }\left(x_{t+1}^{adv}\right)$26:      ${\left(x,y\right)}_{Adv}\leftarrow x_{mim}$27:**end for**28:**return** ${\left(x,y\right)}_{adv}$29:Method: PGD adversarial attack30:Parameter initialisation:31:Set $x_{0}\leftarrow x$;32:Step size: β lies in the range of 0<β≪133:Iteration T← random value from {10,20,30}34:**for** t=0 to t=(T−1) **do**35:      Compute the gradient: $g_{t}\leftarrow \left(\nabla_{x}J\left(\theta ,x_{t},y_{t}\right)\right)$36:      Select the gradient action step: $x_{t+1}^{\prime }\leftarrow x_{t}+\beta \cdot \mathrm{sign}\left(g_{t}\right)$37:      Project ϵ within (ϵ)-ball: $x_{t+1}\leftarrow \Pi_{B\epsilon (x)}\left(x_{t+1}^{\prime }\right)$38:      ${\left(x,y\right)}_{Adv}\leftarrow x_{T}$39:**end for**40:**return** ${\left(x,y\right)}_{Adv}$


The adversarially trained model generated pure samples that are tested using a classifier to check their robustness. The layer-by-layer architecture of the classifier and its description is shown in Table 2. The clean input samples are classified with high accuracy. When adversarial attacks are initiated against the clean samples, the classification accuracy drops. The adoption of adversarial training using the hybrid quantum-classical model purifies the adversarial samples to some extent and increases the classification accuracy.
**Algorithm 2** Adversarial Defence: HQMSGAN Adversarial training algorithm**Require:** Clean samples ${\left(x,y\right)}_{clean}$, adversarial samples ${\left(x,y\right)}_{adv}$ **Ensure:** Purified samples ${\left(x,y\right)}_{pure}$ where $x_{j}\in X$ is the output, $y_{j}\in Y$ is the label.
  1:Parameter initialisation:  2:qubit: *q*, quantum layer: *l*  3:Learning rate: $lr_{g}$, $lr_{d}$; perturbation rate: ϵ; coefficient of gradient penalty: $\lambda_{1},\lambda_{2},\lambda_{3}$  4:Initialise: $G_{Adv_{Loss}}$, $G_{Rec_{Loss}}$, $G_{Class_{Loss}}$, $G_{Fea_{Loss}}$, $G_{Sal_{Loss}}$, $D_{loss_{1}}$, $D_{loss_{2}}$, $D_{loss_{3}}$  5:Adversarial Training Method:  6:Hybrid quantum-classical generator: $\left(G_{\theta G}\right)$  7:Read: ${\left(x,y\right)}_{sample}:{\left(x,y\right)}_{clean}\cup {\left(x,y\right)}_{adv}$  8:**for** all images fed to $\left(G_{\theta G}\right)$ **do**  9:      Encode: $E_{\theta E}{\left(x,y\right)}_{sample}$10:      **for** VQC: ${|\psi \rangle }_{\left(q,l\right)}$) **do**11:            Angle encoding: $|\Psi_{encode}\left(\vec{x}\right)\rangle ={⨂}_{i=1}^{n}R_{Y}\left(\theta_{i}\right)|0\rangle$ where $R_{Y}\left(\theta_{i}\right)=\cos\left(\frac{\theta_{i}}{2}\right)|0\rangle +\sin\left(\frac{\theta_{i}}{2}\right)|1\rangle$12:            **for** l = 1 to n **do**13:                  **for** q = 1 to m **do**14:                        Variational Rotation: $U_{\theta }^{l}$ = $R_{x}\left(\theta_{x}\left[q,l\right]\right)R_{y}\left(\theta_{x}\left[q,l\right]\right)$15:                        Entanglement: $|CNOT\left(x_{i},x_{i+1}\right)\rangle =\left\{\begin{array}{ll} flip\;x_{i+1}, & \mathrm{when}\;x_{i}=1,\\ no\;change, & \mathrm{otherwise}. \end{array}\right.$16:                  **end for**17:            **end for**18:            **for** i = 1 to n **do**19:                  Measure: M(|ψ〉(wires=i))20:            **end for**21:            Return ${M|\psi \rangle }_{\left(q,l\right)}:\left[m_{1},m_{2},m_{3},\ldots ,m_{n}\right]$22:      **end for**23:      Decode: $D_{\theta D}{\left(x,y\right)}_{pure}$24:      $G_{\theta G}$ learns to generate ${\left(x,y\right)}_{sample}^{pure}$25:      $G_{Adv_{Loss}}=-E_{\tilde{x}∼P_{g}}\left[D(\tilde{x})\right]$26:      $G_{Rec_{Loss}}=0.5L_{MSE}+0.5L_{L1}$27:      $G_{Class_{Loss}}=G_{Class_{Loss}}=CE\left(C\left(I_{puresample}\right),y\right)$28:      $G_{\mathrm{FeaLoss}}={∥\phi \left(I_{puresample}\right)-\phi \left(I_{cleansample}\right)∥}_{2}^{2}$29:      $G_{\mathrm{SalLoss}}={∥S_{puresample}-S_{cleansample}∥}_{2}^{2}$30:      $G_{Loss}=0.001G_{Adv_{Loss}}+15G_{Rec_{Loss}}+8G_{Class_{Loss}}+15G_{Fea_{Loss}}+10G_{Sal_{Loss}}$31:**end for**32:Update the generator parameter $\theta_{\theta G_{i}}\leftarrow \theta_{G_{i}}-lr_{g}\nabla_{\theta_{G}}G_{loss}$33:Multi-scale discriminators: $D_{\theta D}\leftarrow D_{\theta D_{1}},D_{\theta D_{2}},D_{\theta D_{3}}$34:Calculate $GP\leftarrow E_{\hat{x}∼P_{\hat{x}}}\left[{\left({∥\nabla_{\hat{x}}D\left(\hat{x}\right)∥}_{2}-1\right)}^{2}\right]$35:**for** all Discriminators in $D_{\theta D}$ **do**36:      Initialise $D_{loss}$37:      Perform multi-scaling on $D_{\theta D}$38:      Downscale $D_{\theta D_{1}}$ to scale: 28×2839:      $D_{loss_{1}}\leftarrow E_{\tilde{x}∼P_{g}}\left[D_{1}\left(\tilde{x}\right)\right]-E_{x∼P_{r}}\left[D_{1}\left(x\right)\right]+\lambda_{1}\;*GP$40:      Downscale $D_{\theta D_{2}}$ to scale: 14×1441:      $D_{loss_{2}}\leftarrow E_{\tilde{x}∼P_{g}}\left[D_{2}\left(\tilde{x}\right)\right]-E_{x∼P_{r}}\left[D_{2}\left(x\right)\right]+\lambda_{2}\;*GP$42:      Downscale $D_{\theta D_{3}}$ to scale: 7×743:      $D_{loss_{3}}\leftarrow E_{\tilde{x}∼P_{g}}\left[D_{3}\left(\tilde{x}\right)\right]-E_{x∼P_{r}}\left[D_{3}\left(x\right)\right]+\lambda_{3}\;*GP$44:**end for**45:D: Learn to discriminate ${\left(x,y\right)}_{adv}$ and ${\left(x,y\right)}_{pure}$: $D_{i}\left(\tilde{x}\right)-D_{i}\left(x\right)$46:$D_{loss}\leftarrow \sum_{i=1}^{3}D_{loss_{i}}$47:Update the discriminator parameter: $\theta_{\theta D_{i}}\leftarrow \theta_{D_{i}}-lr_{d}\nabla_{\theta_{D}}D_{loss}$48:return ${\left(x,y\right)}_{pure}$


## 5. Experiments

The experiment is carried out on the proposed model using both clean samples from the dataset and adversarial samples generated by four techniques: FGSM, BIM, MIM, and PGD. The PennyLane framework, developed by Xanadu, an open-source platform that facilitates quantum computing, is used for implementation. PyTorch version 2.11.0 and Torchvision version 0.26.0 were integrated into the PennyLane based integration. The AWS Braket quantum computing platform offers three quantum simulators, specifically the State Vector 1 (SV1) simulator, Tensor Network 1 (TN1) simulator, and the Density Matrix simulator. The experiments are carried out using the SV1 simulator. The details of the AWS simulators are listed in Table 3.

### 5.1. Benchmark Dataset

The benchmarked MNIST [53] and F-MNIST [54] datasets are used for experimental purposes. MNIST is a dataset containing handwritten character images from 0 to 9 that are grayscale images of size 28×28. The dataset contains 60,000 and 10,000 images for training and testing, respectively. Similarly, the F-MNIST dataset contains fashion products categorised into 10 categories. The ten category images are the images of trousers, pullovers, dresses, coats, sandals, t-shirts, sports shoes, ankle boots, and bags. The division of the dataset into training and testing sets is the same as in the MNIST dataset.

### 5.2. Hyperparameter Setting

To expedite reproducibility, we use the hyperparameters as shown in Table 4 and Table 5 during experimentation. Two datasets, namely the MNIST and Fashion MNIST datasets, are used with 28×28 as the input and output dimensions. For training both the classifiers and the proposed model, the Adam optimiser is used. It enhances faster convergence rate and operates well with sparse gradients. A total of nine configurations of quantum circuits are experimented with a combination of three simulating qubits $q_{4},q_{6},q_{8}$ and three quantum layers $l_{4},l_{6},l_{8}$. The models are trained for 50 epochs with a batch size of 64 for all nine quantum circuit configurations. The $\beta_{1}$ and $\beta_{2}$ values are set to 0.5 and 0.999 within the three down scaling discriminators $D_{1}$, $D_{2}$, and $D_{3}$. When the perturbation rate is very low, for example, when ϵ=0.01,0.001, they are invisible to the human visual system. So we have assumed four perturbation values, ϵ={0.1,0.2}, for state-of-the-art comparison with existing works by Shi et al. [40] and ϵ={0.3,0.5} for comparision with Laykaviriyakul et al. [36].

### 5.3. Ansatz Configuration

An ansatz is a parameterised quantum circuit with tunable parameters. For experimentation purposes, we make use of three input circuits: 4-qubit, 6-qubit and 8-qubit circuits $\left(q_{4},q_{6},q_{8}\right)$ with three different quantum layer patterns: 4, 6 and 8 layers $\left(l_{4},l_{6},l_{8}\right)$. The quantum layers reflect the depth information of the circuit. With the above inputs and layers, there are: a 4-qubit circuit with 4 layers ($q_{4}l_{4}$); a 4-qubit circuit with 6 layers ($q_{4}l_{6}$); a 4-qubit circuit with 8 layers ($q_{4}l_{8}$); Similarly a 6-qubit circuit with 4 layers ($q_{6}l_{4}$), 6 layers ($q_{6}l_{6}$), and 8 layers ($q_{6}l_{8}$); and an 8-qubit circuit with 4 layers ($q_{8}l_{4}$), 6 layers ($q_{8}l_{6}$), and 8 layers ($q_{8}l_{8}$); Thus we get a total of 9 different ansatz configurations.

### 5.4. Ansatz Trainable Parameter Configuration

For the $q_{4}l_{4}$ quantum circuit configuration, the single qubit rotation gates $R_{Y(\theta_{1})}$, $R_{Z(\theta_{2})}$, have 2 distinct parameter count. Since it is a 4 qubit circuit, there is a total of 2×4=8 parameters. There are 4 layers of two rotation gates $R_{X(\theta_{3})}$, $R_{Y(\theta_{4})}$ for a 4-qubit circuit, with a parameter count of 2×4×4=32. There are three entanglemets from $q_{0}$ to $q_{1}$, $q_{1}$ to $q_{2}$ and $q_{2}$ to $q_{3}$. These three CNOT gates do not have any trainable parameters. This sums to a total of 8+32=40 trainable parameters as shown in Table 6. The nine-circuit configuration $q_{i}l_{i}$ is adopted from Shi et al. [40]. Our proposed circuit requires fewer parameters when compared to the parameters required by Shi et al. [40] and much fewer parameters when compared to the classical generator model.

## 6. Results and Discussion

The experimental results of the proposed HQMSGAN model are evaluated by measuring classification accuracy and image quality before and after incorporating adversarial training.

### 6.1. Classification Accuracy

**Figure 4:**
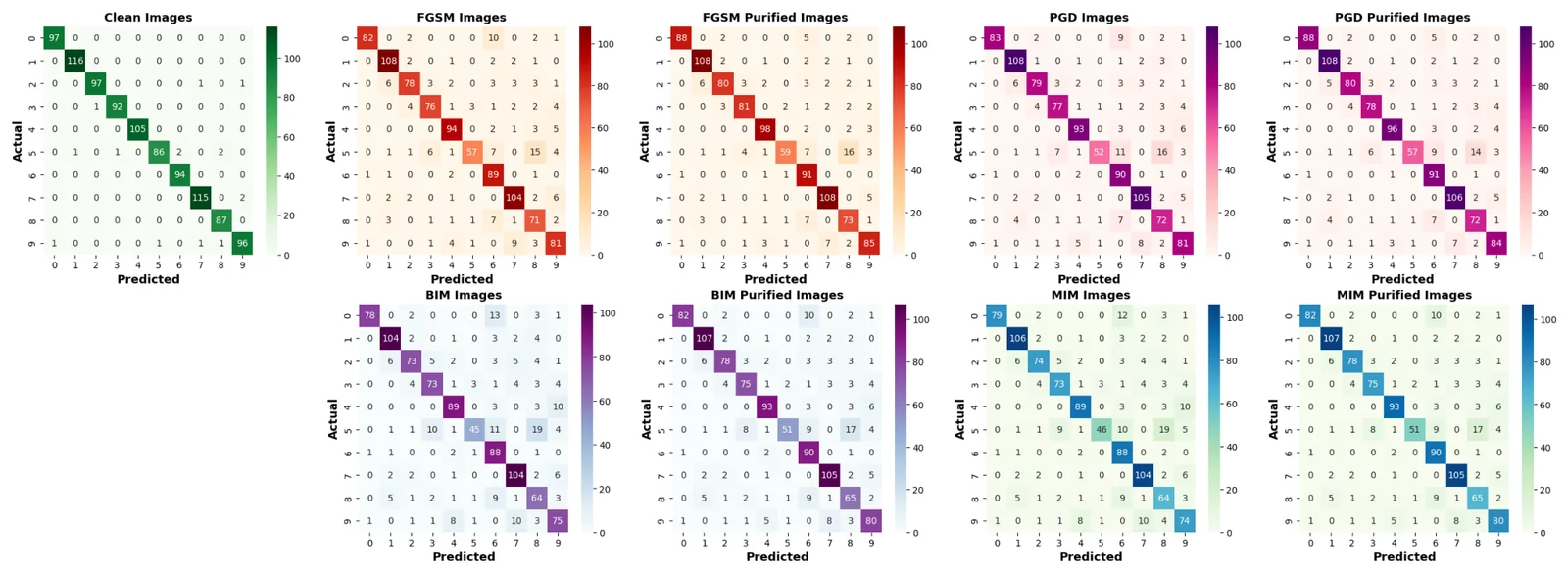
Confusion matrix of clean samples, four adversarial attack samples with perturbation (ϵ=0.3), and their corresponding purified samples for the quantum circuit configuration $q_{8}l_{8}$—MNIST dataset.

**Figure 5:**
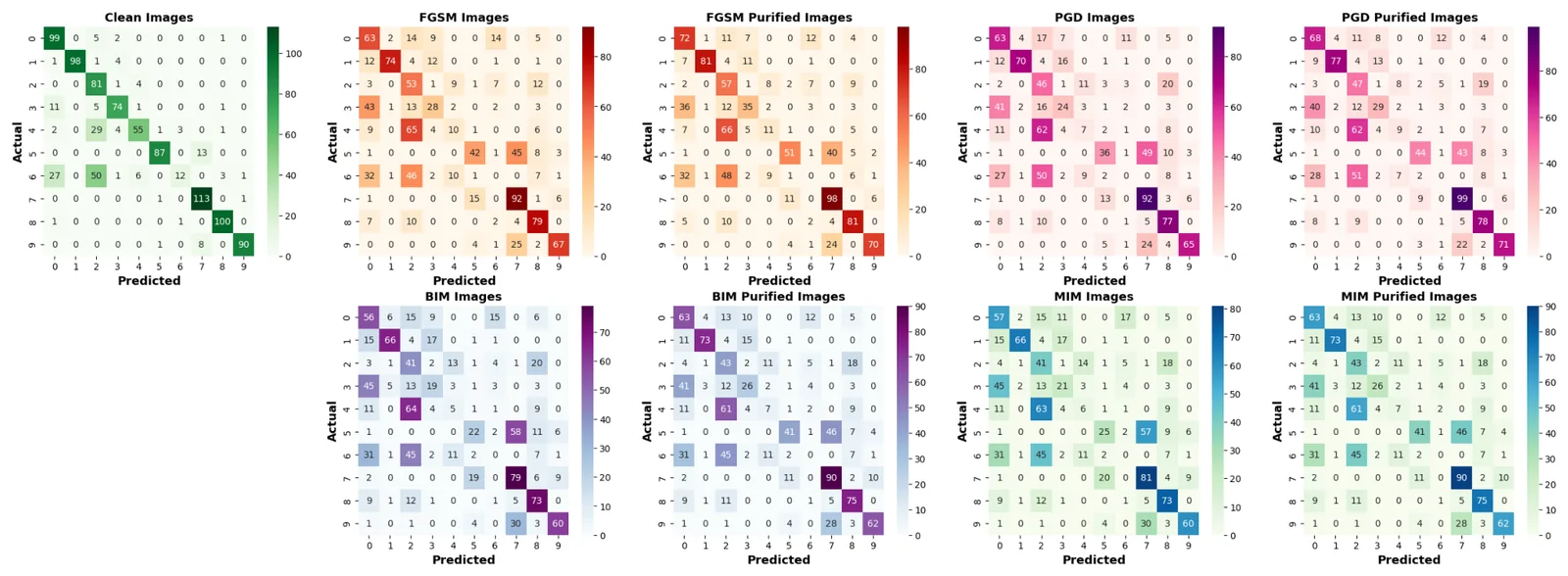
Confusion matrix of clean samples, four adversarial attack samples with perturbation (ϵ=0.3), and their corresponding purified samples for the quantum circuit configuration $q_{8}l_{8}$—Fashion MNIST dataset.

**Figure 6:**
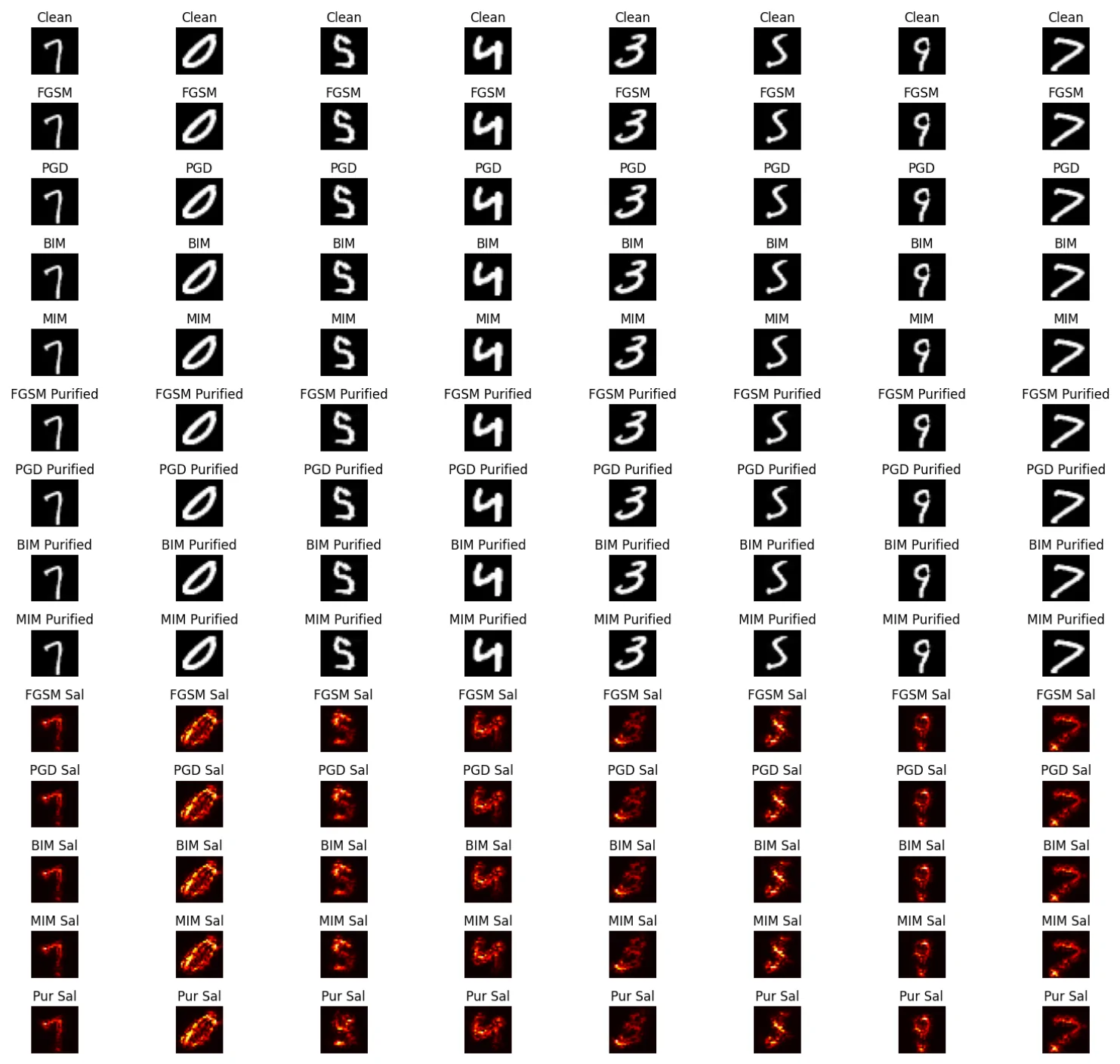
Clean sample, four types of adversarial samples with their respective purified samples and their saliency maps for ϵ=0.01—MNIST dataset.

**Figure 7:**
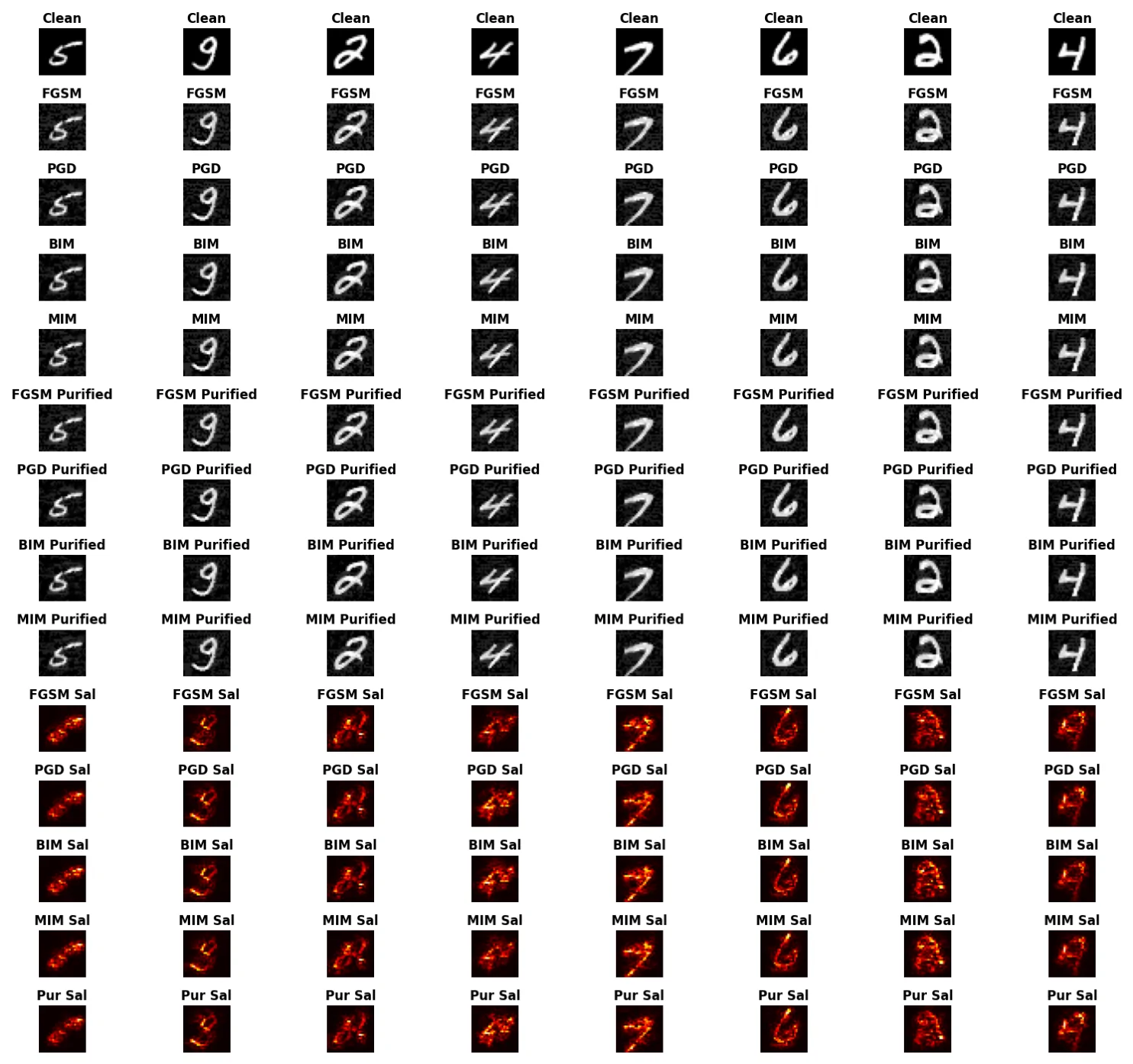
Clean sample, four types of adversarial samples with their respective purified samples and their saliency maps for ϵ=0.2—MNIST dataset.

**Figure 8:**
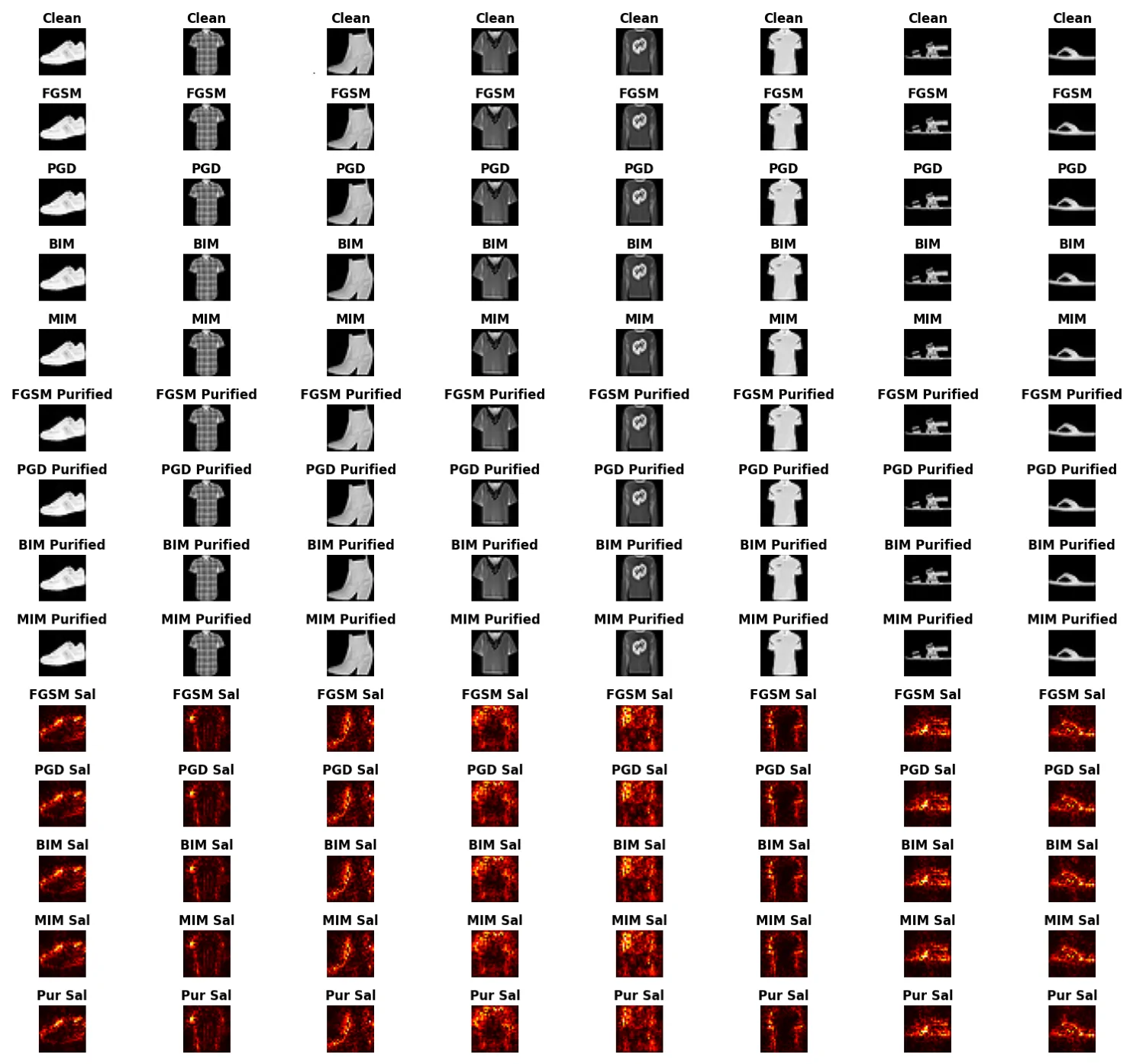
Clean sample, four types of adversarial samples with their respective purified samples and their saliency maps for ϵ=0.01—Fashion MNIST dataset.

**Figure 9:**
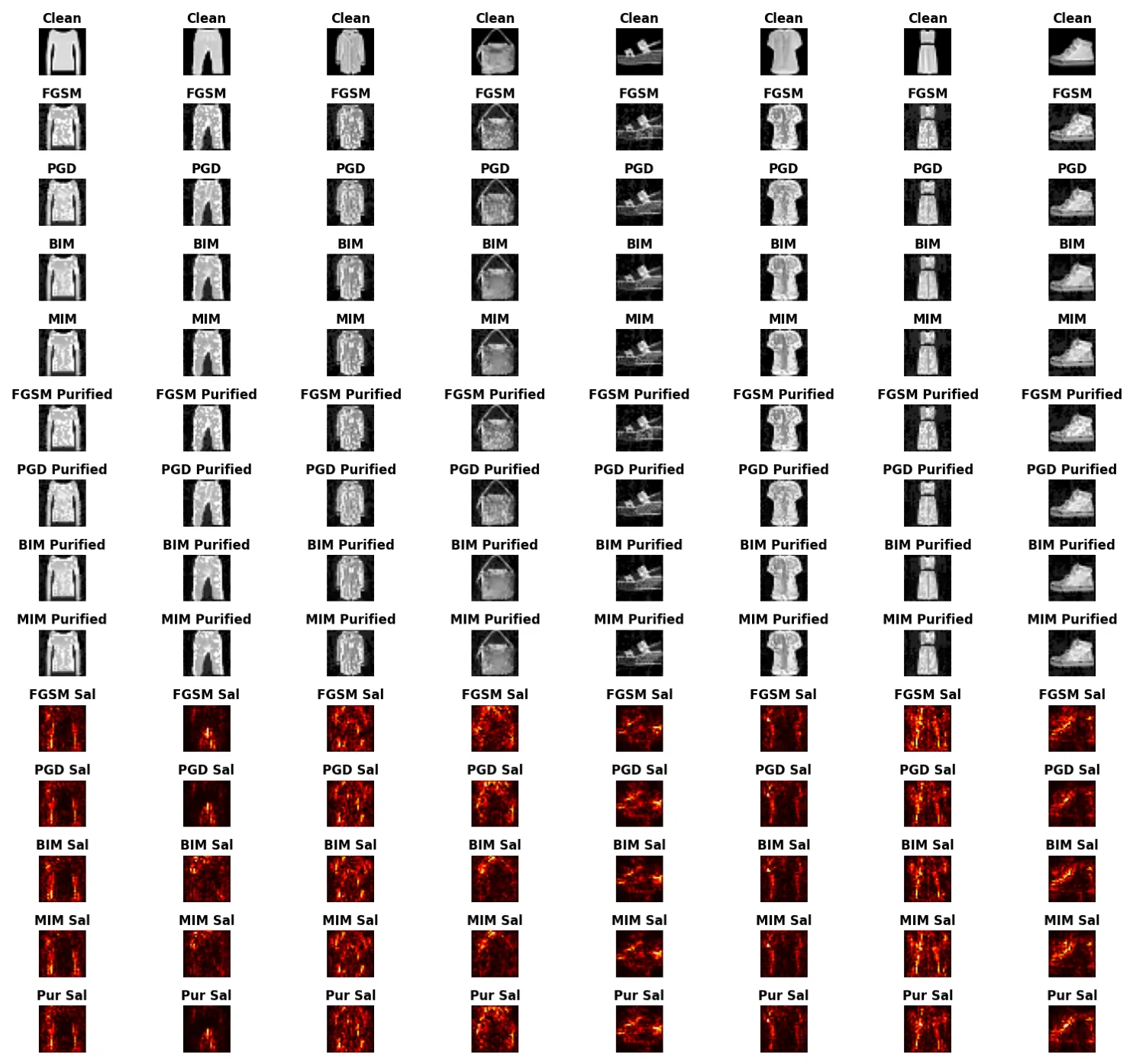
Clean sample, four types of adversarial samples with their respective purified samples and their saliency maps for ϵ=0.5—Fashion MNIST dataset.

**Figure 10:**
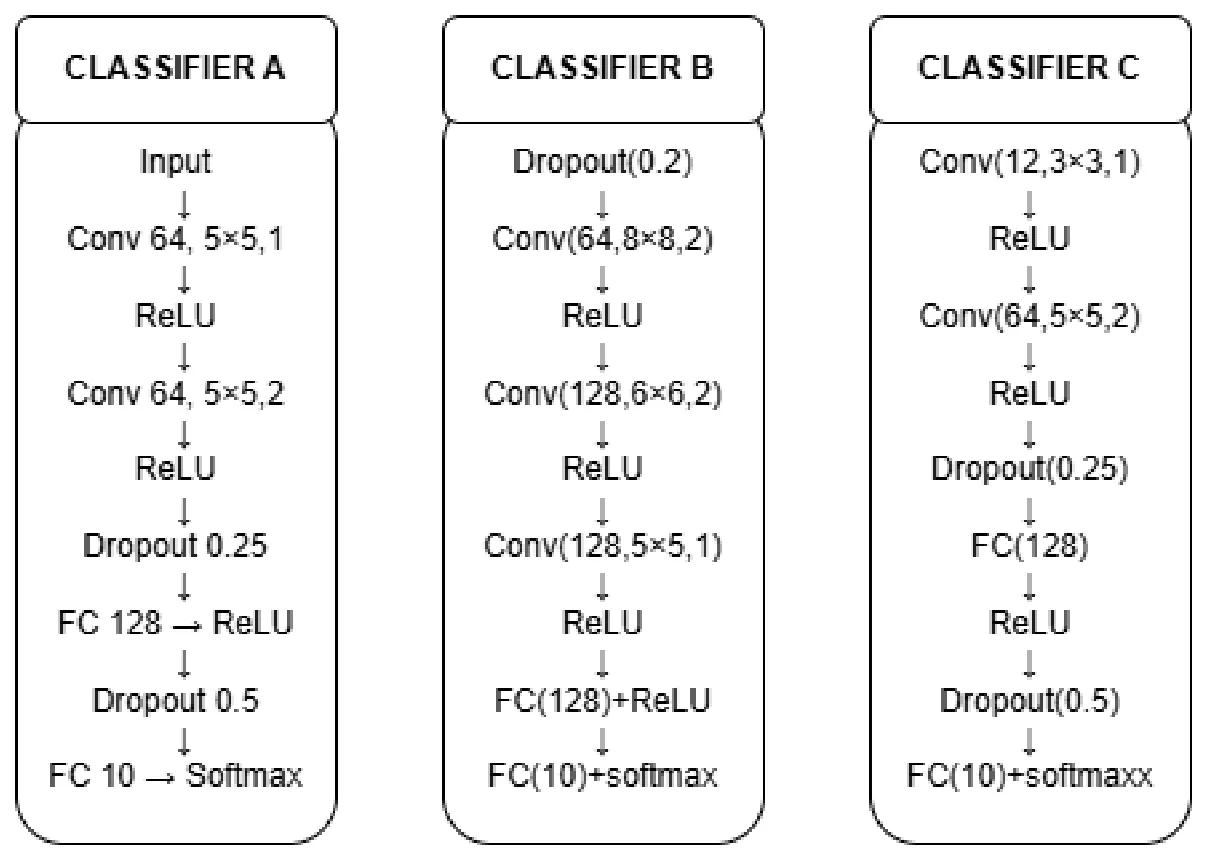
The model architecture of the three classifiers A, B, and C adopted from Laykaviriyakul et al. [36] used for tabulation of values in Table 10 and Table 11.

The classification accuracy is evaluated using the confusion matrix. The classification accuracy of the clean data, adversarially attacked data, and adversarially trained data are evaluated. The confusion matrix displays the comparison of predicted values against actual values. It is printed to differentiate between diverse classes of the dataset. As the dataset contains 10 classes, the confusion matrix is a 10×10 matrix, and the class-level performance can be visualised in the diagonal elements. The diagonal elements in the clean image classification display high values, indicating correct classification. This performance degrades in the other confusion matrices due to the presence of adversarial perturbations. After adversarial training, the confusion matrix is printed again to visualise the changes after purification. The confusion matrices for the clean samples, four adversarial attack samples, and their corresponding adversarial defence samples for MNIST and Fashion MNIST are shown in Figure 4 and Figure 5, respectively. The classification accuracy on the clean, adversarially perturbed MNIST, and Fashion MNIST datasets for nine quantum circuit configurations of $q_{i}l_{i}$, where i=4,6,8, is shown in Table 7 and Table 8, respectively. The average clean accuracy for the MNIST and FashionMNIST datasets is 98.04% and 81.23%, respectively. But when the four kinds of adversarial attacks are launched, the accuracy values drop drastically. By deploying the adversarial training strategy, the defence accuracy is increased. The clean and adversarial samples of the MNIST datasets when the perturbation rate is ϵ=0.01,0.2 are shown in Figure 6 and Figure 7, respectively. The clean and adversarial samples of the Fashion MNIST datasets when the perturbation rate is ϵ=0.01,0.5 are shown in Figure 8 and Figure 9, respectively. The clean samples, attack samples, purified samples and saliency map are shown in the figures. The saliency map shows the most prominent area of the image is generated to find out the visual prominence of the image. This helps to determine whether the meaningful features of the images are restored once the images are purified after incorporating the adversarial training defence framework. Table 9 shows the attack rate comparison with Shi et al. [40] when ϵ values are 0.1 and 0.2. Table 10 and Table 11 show the comparison of classification accuracy for the MNIST and FashionNIST dataset with related works such as MagNet [33], DefenseGAN [34], and Collaborative defence GAN [36] with adversarial training parameter ϵ=0.3. Three classifier architectures are adopted from Laykaviriyakul et al. [36] for comparison of results. The architectures of the three classifier models A, B, and C are shown in Figure 10. On comparison, it is found that the proposed method has outperformed the existing works to the greatest extent possible.

### 6.2. Measurement of Image Quality

The image quality of the purified samples is assessed using four metrics, namely the SSIM, PSNR, FID and LPIPS. The SSIM value is computed by analysing the changes in the image by finding the luminance, contrast, and structural details of the image. SSIM values that are closer to 1 show a good correlation value. The PSNR measures the pixel-by-pixel differences in data points of the image or the pixel-by-pixel math error, while SSIM evaluates human perceptual structural changes like brightness and contrast. The SSIM is calculated using the three most important visual parameters, namely, luminance, contrast, and structural details of the image.

(26) $$SSIM\left(a,b\right)=\frac{\left(2\mu_{a}\mu_{b}+C_{1}\right)\left(2\sigma_{ab}+C_{2}\right)}{\left(\mu_{a}^{2}+\mu_{b}^{2}+C_{1}\right)\left(\sigma_{a}^{2}+\sigma_{b}^{2}+C_{2}\right)}$$

(27) $$\mathrm{MSE}=\frac{1}{mn}\sum_{i=1}^{m}\sum_{j=1}^{n}{\left(Clean_{\left(i,j\right)}-Purified_{\left(i,j\right)}\right)}^{2}$$

**Figure 11:**
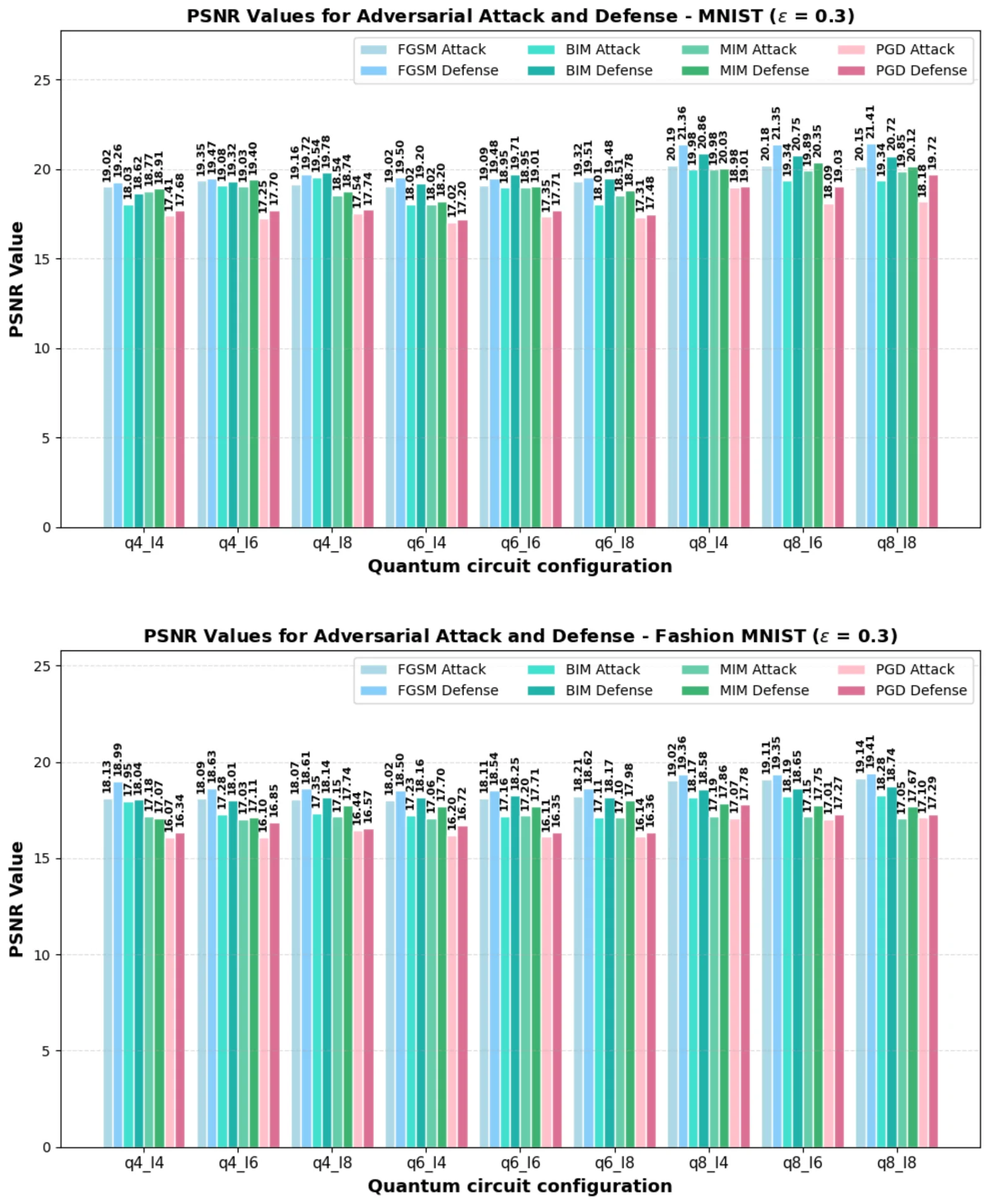
Measurement of image quality of the four adversarial attack and defence images by measuring the PSNR value using nine quantum circuit configurations—MNIST and Fashion MNIST dataset when ϵ=0.3.

**Figure 12:**
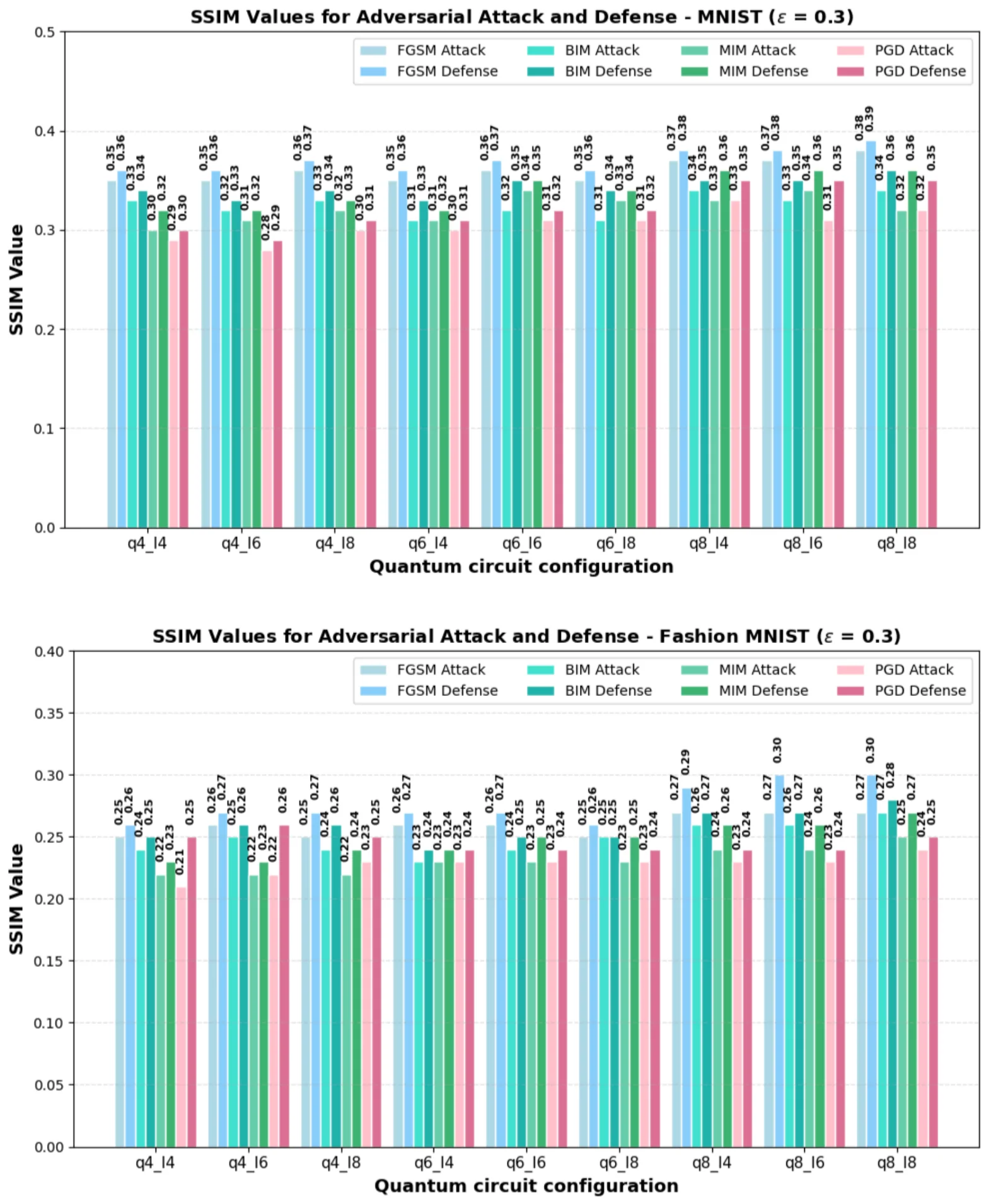
Measurement of image quality of the four adversarial attack and defence images by measuring the SSIM value using nine quantum circuit configurations—MNIST and Fashion MNIST dataset when ϵ=0.3.

The SSIM metric is computed using the local mean $\mu_{a},\mu_{b}$, the standard deviation $\sigma_{a},\sigma_{b}$, and the cross-covariance $\sigma_{ab}$. The regularisation constants $C_{1},C_{2}$, and $C_{3}$ are added to address the instability issues caused when the local mean or standard deviation value reduces and moves close to zero. The PSNR value is computed by finding the pixel-wise difference between the clean sample and the reconstructed adversarially trained sample. It is computed using the Mean Squared Error (MSE) as follows:

(28) $$\mathrm{PSNR}=10\log_{10}\left(\frac{MAX_{I}^{2}}{\mathrm{MSE}}\right)$$

where $MAX_{I}$ represents the pixel value of the image sample. For the MNIST and Fashion MNIST datasets, the pixel value ranges from 0 to 255, so $MAX_{I}=255$. The image quality of the four adversarial attack and defence images is measured for the MNIST and Fashion MNIST datasets when ϵ=0.3 using the PSNR value and the SSIM value from the nine quantum circuit configurations. The graph of the PSNR values is shown in Figure 11. The graph of the SSIM values are shown in Figure 12. From these two graphs, it is evident that the adoption of adversarial training improved the similarity scale across all quantum configurations.

**Figure 13:**
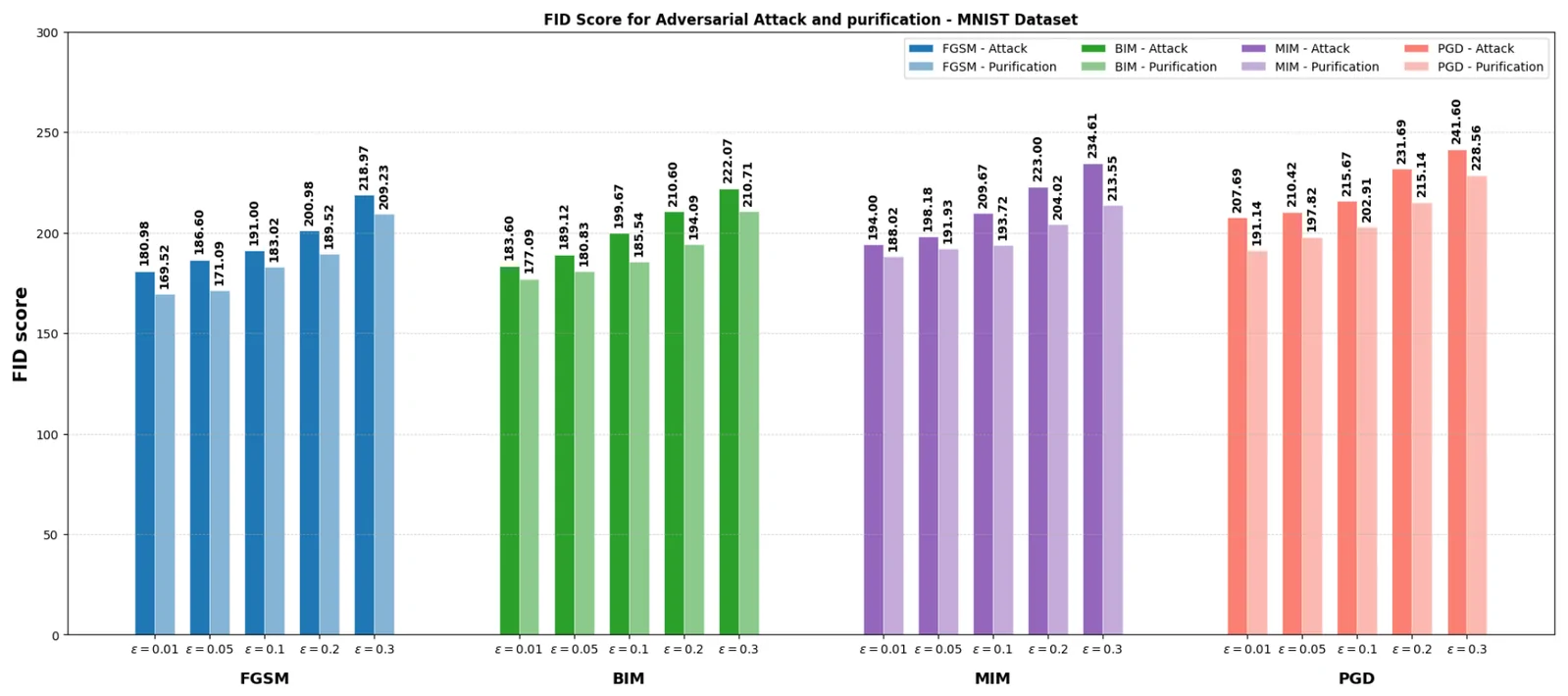
Measurement of image quality of the four adversarial attack and purification images by measuring the FID score using ϵ=0.01,0.05,0.1,0.2,0.3 values for the MNIST dataset.

**Figure 14:**
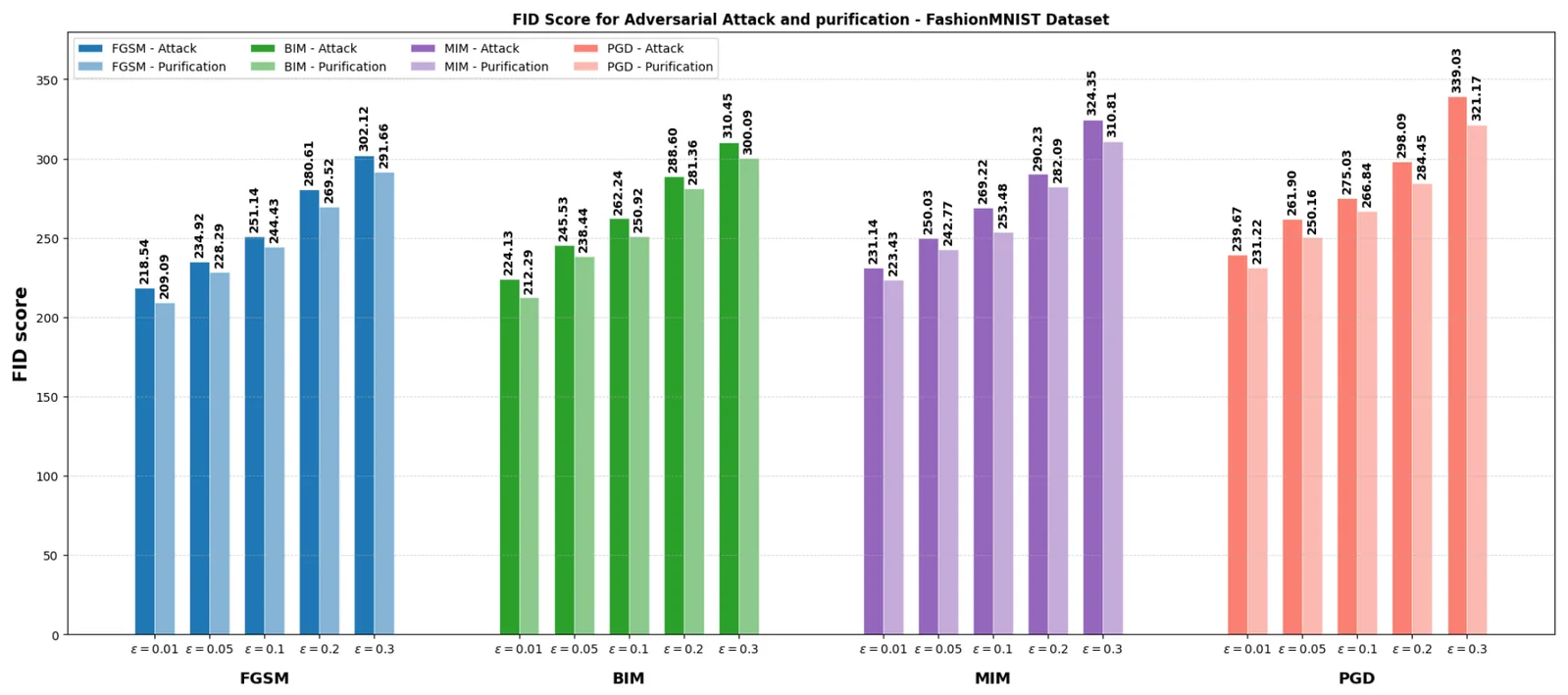
Measurement of image quality of the four adversarial attack and purification images by measuring the FID score using ϵ=0.01,0.05,0.1,0.2,0.3 values for the FashionMNIST dataset.

The FID score measures the difference between the probability distributions of two different image sets. The lower the FID score, the better the generated image. The difference in similarity between the clean dataset and the adversarial attack dataset, and the difference in similarity between the clean dataset and the purified dataset, are measured. The FID score across five different perturbation rates, ϵ=0.01,0.15,0.1,0.2,0.3, is plotted for the MNIST and FMNIST datasets and shown in Figure 13 and Figure 14, respectively. The purified samples have a lower FID score than the adversarial attack samples.

**Figure 15:**
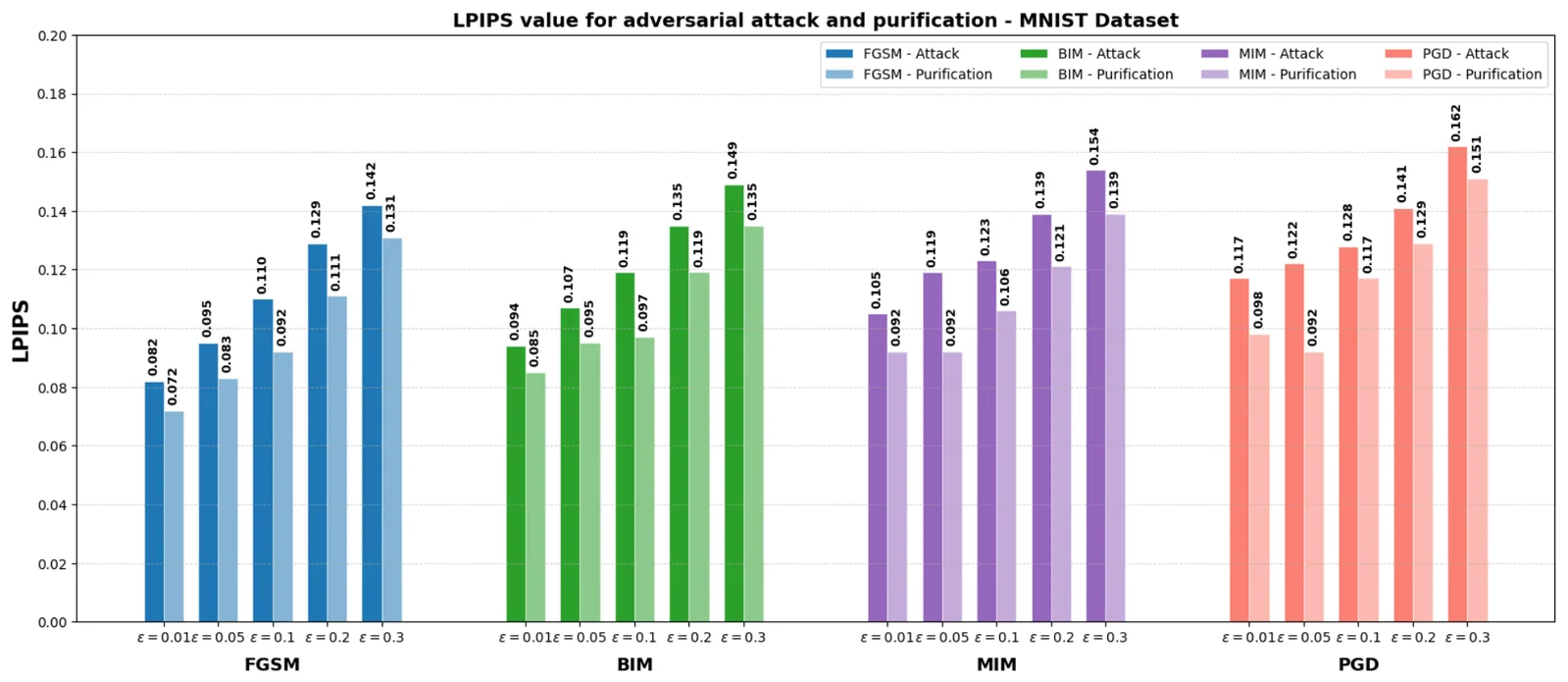
Measurement of image quality of the four adversarial attack and purification images by measuring the LPIPS value using ϵ=0.01,0.05,0.1,0.2,0.3 values for the MNIST dataset.

**Figure 16:**
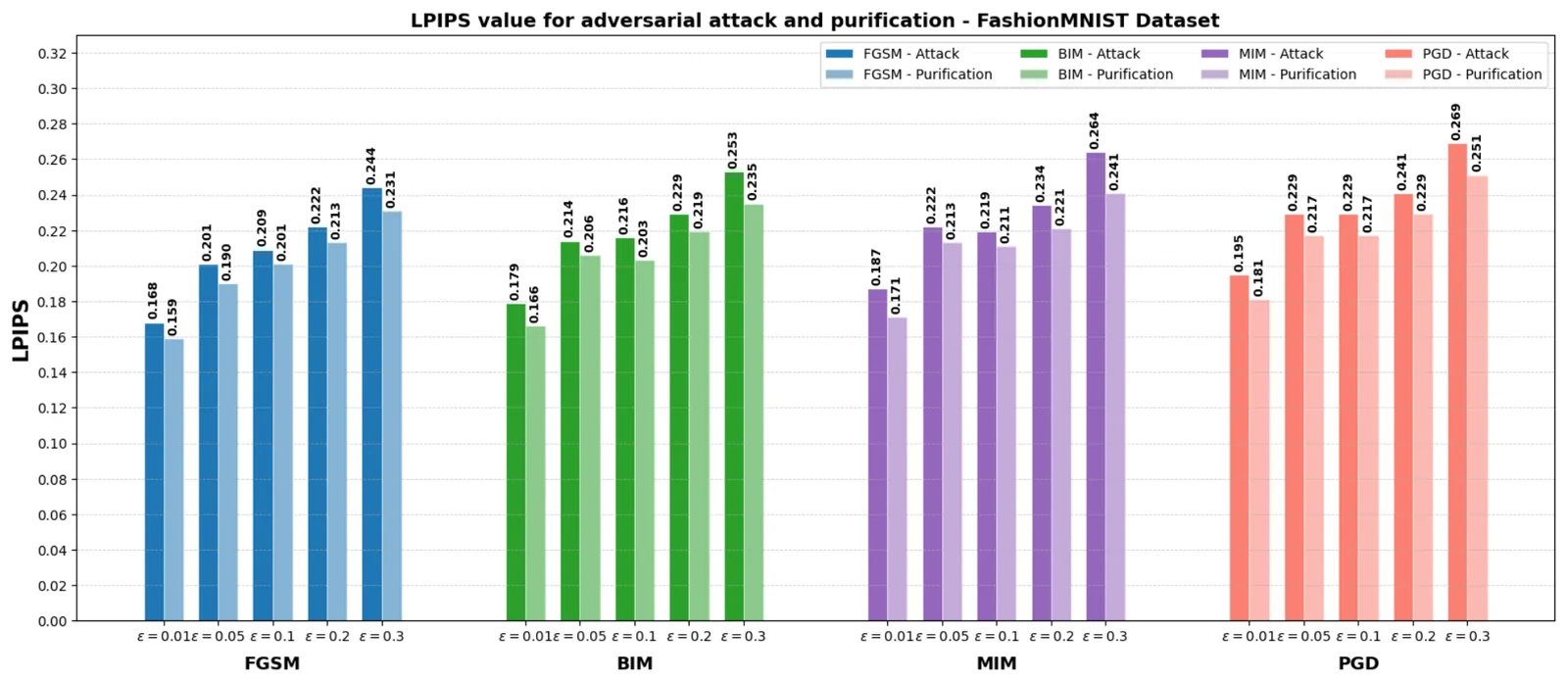
Measurement of image quality of the four adversarial attack and purification images by measuring the LPIPS value using ϵ=0.01,0.05,0.1,0.2,0.3 values for the FashionMNIST dataset.

The LPIPS metric is a perceptual similarity metric used to measure the distance between the clean sample and the purified sample. The LPIPS value across five different perturbation rates, ϵ=0.01,0.15,0.1,0.2,0.3, is plotted for the MNIST and FMNIST datasets and shown in Figure 15 and Figure 16, respectively. The lower the LPIPS score, the higher the perceptual similarity.

### 6.3. Evaluation of Loss

**Figure 17:**
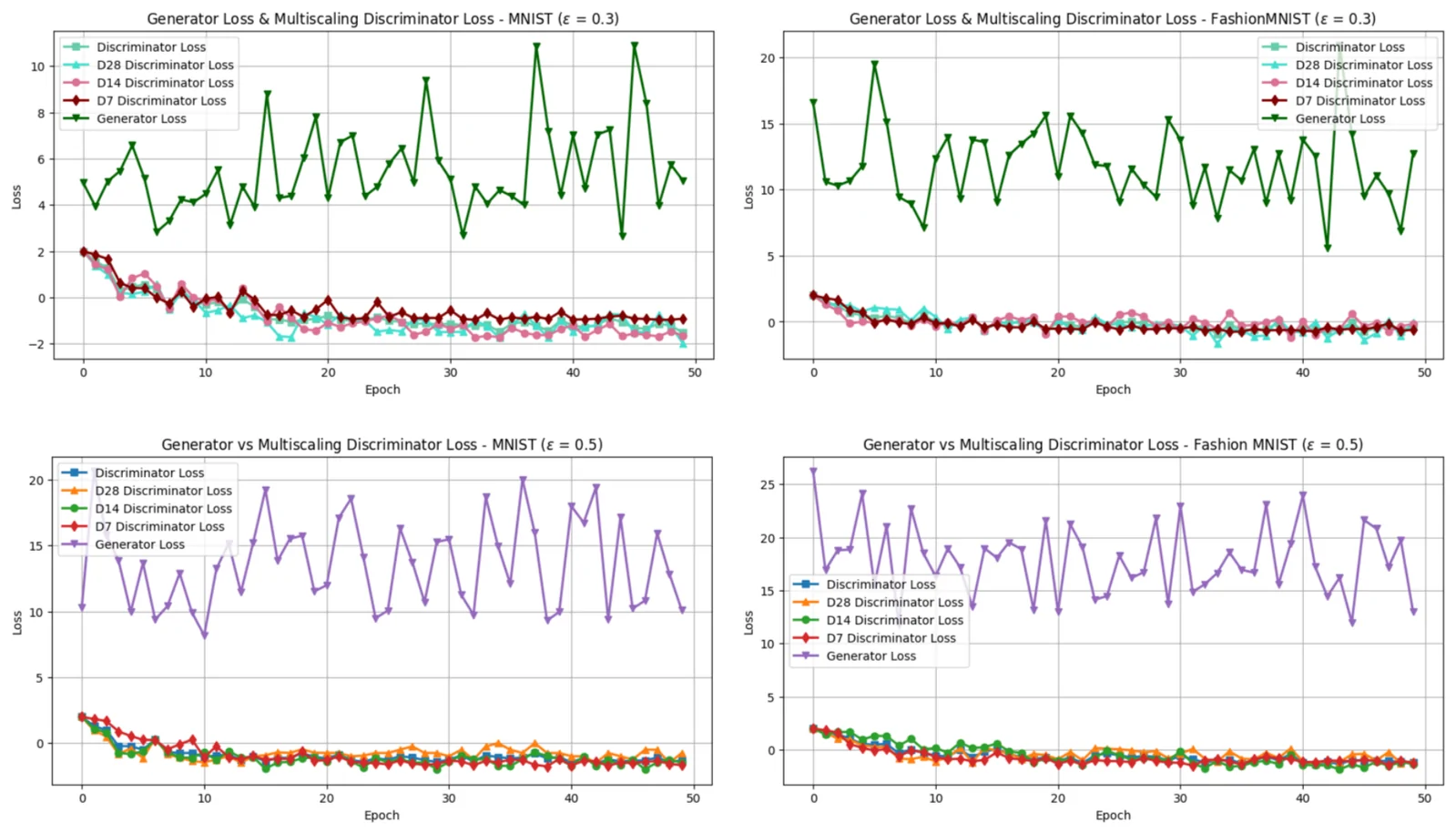
Loss of the generator and multiscaling discriminator of the $q_{6}l_{4}$ quantum circuit configuration for the MNIST and FMNIST datasets when ϵ=0.3,0.5.

**Figure 18:**
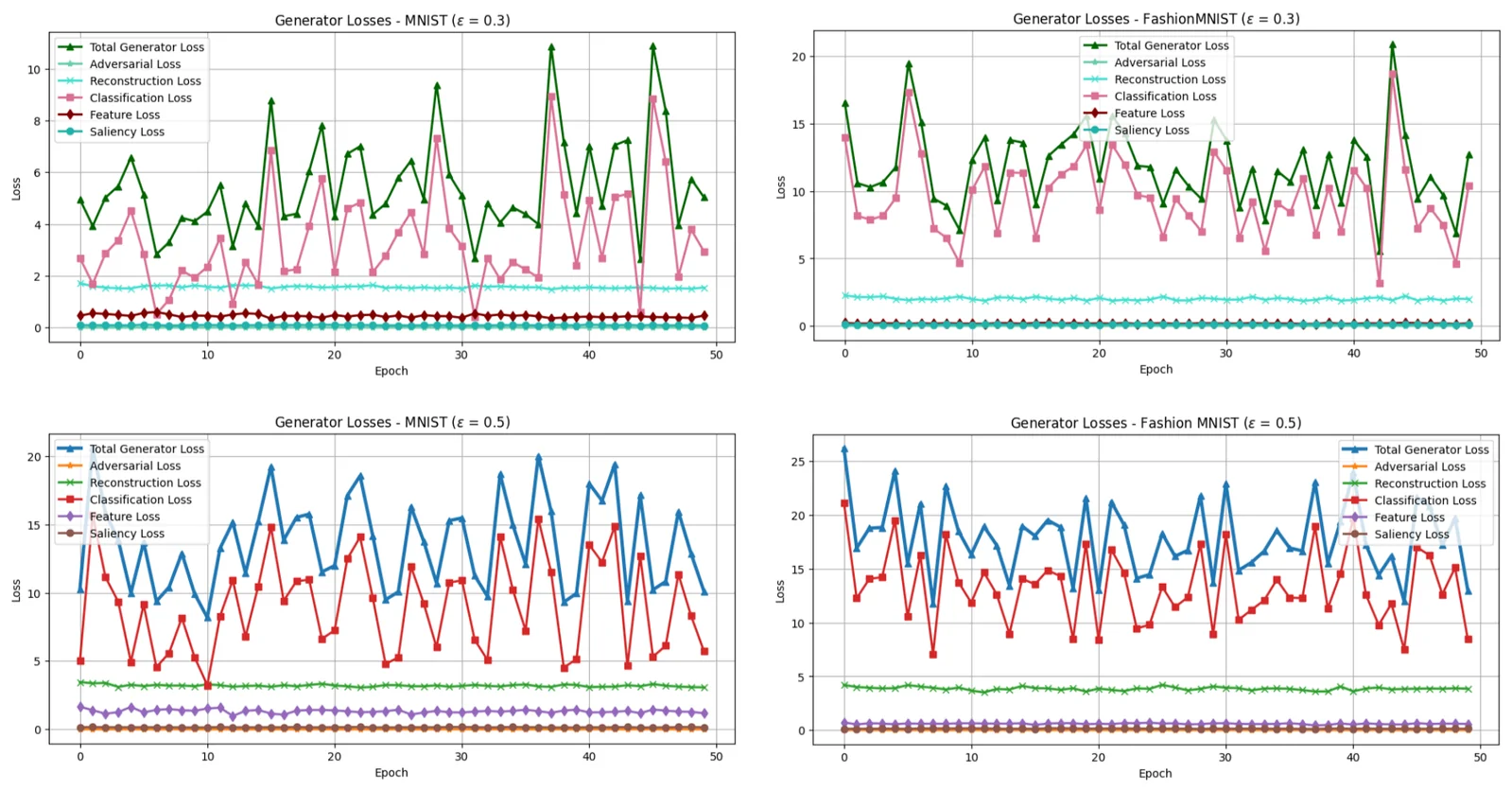
The five losses along with the total loss experienced by the quantum generator of the $q_{6}l_{4}$ quantum circuit configuration for the MNIST and FMNIST datasets when ϵ=0.3,0.5.

The loss experienced by the generator and discriminator of the proposed model is computed for the MNIST and F-MNIST datasets. The loss of the generator and multiscaling discriminator of the $q_{6}l_{4}$ quantum circuit configuration for the MNIST and FMNIST datasets when ϵ=0.3,0.5, measured across 50 epochs, is shown in Figure 17. It shows the overall generator loss and the multiscaling loss of three discriminators of scale $D_{1}=28\times 28$, $D_{2}=14\times 14$, $D_{3}=7\times 7$. The generator experiences five losses, namely, the adversarial loss, the reconstruction loss, the classification loss, the feature loss and the saliency loss. These losses along with the total loss are measured for the $q_{6}l_{4}$ quantum circuit configuration and shown in Figure 18.

### 6.4. Ablation Study

This section deals with the analysis and study of various experimental factors that influence the output.

#### 6.4.1. Ablation Study on Qubits and Quantum Layers

The ablation study on the quantum circuit was done by altering the qubit count and depth of the quantum circuit. When the qubit count and layer count are small, for example, a $q_{4}l_{4}$ circuit results in poor performance. With the increasing number of qubits, the Hilbert space grows progressively. Higher qubit count and deeper quantum layers facilitate better feature mapping. The qubit entanglement expedites latent correlations among features. This in turn facilitates inter-feature coupling in vast dimensional spaces, resulting in better robustness against adversarial attacks. It is evident from Table 7 and Table 8 that the quantum circuit with 8 qubits, 6 quantum layers, $q_{8}l_{6}$, and 8 qubits, 8 quantum layers, $q_{8}l_{8}$, have obtained better results for accuracy and similarity index when compared to lower dimensional circuits.

#### 6.4.2. Ablation Study on Attack and Defence Accuracy

The study on the clean data accuracy, attack and defence accuracy is analysed using data from Table 7 and Table 8. For the MNIST dataset, the average clean data accuracy is 98.07%. When a perturbation rate of ϵ=0.3 is applied, the average accuracy drops to 84.94% when the FGSM attack is launched. By performing adversarial training, this average accuracy increases to 86.60%. There is an absolute improvement of 1.66%. Similarly, the average accuracy of the BIM attack rises from 83.15% to 85.07%, an absolute improvement of 1.91%, the MIM attack rises from 78.33% to 81.27%, an absolute improvement of 2.94%; the PGD attack rises from 69.35% to 71.92%, an absolute improvement of 2.56%. For the same dataset, when the perturbation rate is ϵ=0.5, the clean data accuracy is 97.79%. The average accuracy drops to 73.04% when the FGSM attack is instantiated. The defence strategy increases the accuracy to 75.15%, resulting in an absolute improvement of 2.11%. Similarly, there is an increase of 1.96%, 1.44% and 1.78% for BIM, MIM, and PGD adversarial defence mechanisms. A similar improvement is seen in the FashionMNIST dataset too.

#### 6.4.3. Ablation Study on Generator Models

The quantum generator model was replaced with a classical generator module to compare its effectiveness. The classical generator model adopted the same design as the proposed model except for the change in the quantum circuit. The VQC was replaced with a neural network with linear and ReLU layers. The performance of the classical model was evaluated using performance metrics like attack accuracy, defence accuracy, SSIM and PSNR values of attack and defence methods. The performance values of the four attacks and their corresponding defence are tabulated in Table 12. When the results are compared with the performance metrics of the hybrid quantum model, as shown in Table 7 and Table 8, the hybrid model excels in performance in terms of accuracy and similarity metrics.

#### 6.4.4. Ablation Study on Multiscaling Discriminators

This section examines the use of multiscaling discriminators for effective training and adversarial robustness. First, the study was done by employing all three discriminators, $D_{1}$, $D_{2}$, and $D_{3}$. It was repeated by employing two discriminators, $D_{1}$, and $D_{2}$, followed by $D_{1}$ alone, and the results were recorded. The changes in accuracy and the structural similarity metrics with varying discriminator count are shown in Table 13 and Table 14 respectively. It is evident from the table that the results incorporating the three multiscaling discriminators excelled in performance when compared to double and single discriminators.

#### 6.4.5. Ablation Study on Loss

This section examines the study of the five loss components involved in during adversarial purification. Each loss is neglected, and the corresponding accuracy of the FGSM adversarial attack is measured. The ablation study on loss is shown in Table 15. The quantum circuit adopted was $q_{4}l_{4}$ on MNIST dataset with 0.3 as the ϵ value. The variation in loss components shows that each loss affects the classification accuracy.

### 6.5. Research Challenges and Future Directions

The HQMSGAN is a hybrid integration of a quantum generator and the classical discriminators. It relies on classical computation of encoders, decoders, and multiscaling discriminators. The generator module performs quantum computation, where qubits are fed into quantum circuits with varying numbers of layers. The strength of the HQMSGAN relies on quantum feature mapping, which diminishes the effect of adversarial perturbations. CNOT gates introduce entanglement, enabling strong interaction and feature coupling among qubits. The hybrid quantum-classical model enhances adversarial robustness by learning the spatial relationships between pixels using quantum entanglement. It performs entanglement to update the correlating pixels simultaneously. It performs angle rotations and embedding to generate samples with precise shades. The quantum properties make the model robust against three white-box adversarial attacks in the quantum realm: FGSM, BIM, and MIM. However, based on the results tabulated in Table 7 and Table 8, the proposed model has given a marginal improvement for the PGD attack. For the generation of high-quality samples, higher-order qubits are required, which is more challenging and expensive. Our proposed model has employed a predefined ansatz structure with varying quantum bits and quantum layers, which is a heuristically designed quantum circuit. However, recent research in quantum computing has thrown light on automating quantum circuit optimization, where the qubit count, quantum layer depth, gate configuration, and entanglement structure are systematically determined using the Quantum Architecture Search (QAS) method [55]. This process is done by a self-supervised machine learning technique that serves as an alternative to the preconfigured quantum circuit in the proposed framework. Similar work has been proposed by Su et al. [56], Li et al. [57], and Ruiz et al. [58], where optimised quantum circuits are employed instead of using a predefined ansatz structure.

## 7. Conclusions

In this paper, we have proposed HQMSGAN, a novel hybrid QGAN-based adversarial training strategy that enhances robustness against adversarial attacks. The proposed methodology is experimented with the MNIST and Fashion MNIST datasets with varying perturbation rates across nine different quantum circuit configurations by varying the qubit count and the quantum layer count. The experiments using the HQMSGAN model have defended against the four adversarial attacks, namely the FGSM attack, BIM attack, MIM attack, and PGD attack, by leveraging quantum generative learning. The multi-scale discriminators have enhanced the image quality of the purified samples and provided training stability of the hybrid quantum GAN. Experimental results show that there is performance improvement in terms of adversarial robustness. The adversarial samples, the purified sample and the saliency map of the sample are generated to visualise the differences in pixel changes. The saliency map identifies the most influential regions contributing to classification. Repeated experimentation on different quantum circuit configurations using quantum simulators was exhaustive, time-consuming and costly. In the future, we intend to study new and strong adversarial attacks using an increased qubit count and quantum layers. The qubit count can be increased to take full advantage in the quantum realm. Future quantum workflows will execute quantum circuits directly on hardware resources provided by AWS Braket, like IonQ and Rigetti, to measure the expectation values. A study with a more complex quantum circuit that facilitates better Hilbert space mapping and instantaneous feature learning and coupling is in the pipeline. Our discoveries to enhance the adversarial robustness against adversarial attacks using the proposed hybrid quantum-classical model with multi-scale discriminators employing different circuit configurations can open a brand-new research direction in the field of quantum adversarial machine learning to study other white-box and black-box attacks.

## Figures and Tables

**Table 1:** Study of related work.

Author	Model	Dataset	Adversarial Attack	Metrics	Inference
Tramer et al. [32]	ResNet	MNIST, ImageNet	FGSM, I-FGSM, PGD	Accuracy, Error rate	The ensemble adversarial training approach improves the diversity of perturbations but slows down the convergence speed and lowers its performance.
Meng et al. [33]	Auto Encoder	MNIST, CIFAR10	FGSM, Iterative, Deepfool, CW	Accuracy	Autoencoder-based detector-reformer model that records 0% accuracy for some defence. Their model is not able to defend against powerful attacks. There is still a requirement for architectural refinement.
Samangouei et al. [34]	GAN	MNIST, FashionMNIST	FGSM, CW	Accuracy	A baseline defence model used for comparison. They face challenges while tuning the sensitive parameters and while training the WGAN model.
Kehoe et al. [35]	QRBM	MNIST	FGSM, DeepFool, CW	Accuracy	A quantum model-based defence technique. The input 28×28 images need to be rescaled to 7×7 before proceeding with the defence. The quantum circuit configuration adopted for experimentation is lacking.
Laykaviriyakul et al. [36]	Disco GAN	MNIST, FashionMNIST, CIFAR-10	FGSM, PGD, CW	Accuracy	A collaborative GAN-based defence technique that employs noise filtering and an adversarial training technique using complex models that slow down the reconstruction of pure images. They incur additional cost while reconstructing the adversarial images.
Baytas et al. [37]	GAN	CIFAR-10, CIFAR-100, SVHN, TinyNet	FGSM, PGD, CW	Accuracy, Loss	A GAN-based model is used to generate perturbation. Their defence strategy is not able to defend against the PGD attack. The GAN model incurs increased training time.
Zhao et al. [38]	DNN	CIFAR-10, CIFAR-100, Tiny ImageNet	PGD, MI-FGSM, CW, AA	Accuracy	The multi-guided adversarial training faces difficulty in balancing decision boundaries. They also face overfitting issues.
Song et al. [39]	GAN	MNIST, CIFAR-10	FGSM, PGD, One Pixel, Deep Fool, CW	Error rate	Defence is done by a denoising technique, using a complex model training procedure that consumes time and effort. Classifier accuracy and similarity metrics to measure the quality of the defence image are missing.
Shi et al. [40]	QCNN	MNIST, Fashion-MNIST	FGSM, BIM, PGD, MIM, CW	Accuracy	A quantum-based defence technique using the QCNN model to defend against adversarial attacks. They have done analysis using nine configurations of quantum circuits. They have asserted that they have done a comparison with four classical models. But the comparison is not highlighted.
Proposed	HQMSGAN	MNIST, FashionMNIST	FGSM, BIM, MIM, PGD	Accuracy, SSIM, PSNR, FID, LPIPS	A hybrid quantum-classical WGAN-based multi-scaling approach to purify the images that are being affected by adversarial attacks using adversarial training. The hybrid quantum-classical part is explored using different qubit weights and quantum layer counts.

**Table 2:** Layer-by-Layer architecture of the classifier.

Layer	Details	Output Dimension
Conv1	Conv(32, 3 × 3, 1), ReLU	32 × 28 × 28
Pooling	Maxpooling(2)	32 × 14 × 14
Conv2	Conv(64, 3 × 3, 1), ReLU	64 × 14 × 14
Pooling	Maxpooling(2)	64 × 7 × 7
Flatten	Flatten	64 × 7 × 7
FC 1	FC1(Linear), ReLU	128
FC2	FC2(Linear)	10

**Table 3:** AWS Braket access providers.

Simulator	Qubits	Availability	Device ARN	Description
SV1	34	24/7	arn:aws:braket:::device/quantum-simulator/amazon/sv1	34 qubit Amazon Braket state vector simulator
TN1	50	24/7	arn:aws:braket:::device/quantum-simulator/amazon/tn1	50 qubit Amazon Braket tensor network simulator
DM1	17	24/7	arn:aws:braket:::device/quantum-simulator/amazon/dm1	17 qubit Amazon Braket density matrix simulator

**Table 4:** Hyperparameter settings.

Parameter	Value	Parameter	Value
Input dimension	28×28	Output dimension	28×28
Simulating qubits	4, 6, 8	Circuit depth	4, 6, 8
Generator learning rate ($l_{g}$)	0.0001	Discriminator learning rate ($l_{d}$)	0.00005
$\beta_{1}$	0.5	$\beta_{2}$	0.999
Batch size	32, 64	Optimiser	Adam

**Table 5:** Parameter settings of adversarial attack types.

Attack Method	Parameter
FGSM attack	ϵ=0.01,0.05,0.1,0.2,0.3,0.5
BIM attack	ϵ=0.01,0.05,0.1,0.2,0.3,0.5, α=0.03, steps = 10
MIM attack	ϵ=0.01,0.05,0.1,0.2,0.3,0.5, α=0.03, μ=1.0, steps = 10
PGD attack	ϵ=0.01,0.05,0.1,0.2,0.3,0.5, α=0.03, steps = 10

**Table 6:** Parameter count for the nine quantum circuit configurations. It is compared with the parameter count of Shi et al. [40].

Circuit Configuration	Qubit Count (q)	Layer Count (l)	Parameters [40]	Proposed Parameters
$q_{4}l_{4}$	4	4	48	40
$q_{4}l_{6}$	4	6	72	56
$q_{4}l_{8}$	4	8	96	72
$q_{6}l_{4}$	6	4	72	60
$q_{6}l_{6}$	6	6	108	84
$q_{6}l_{8}$	6	8	144	108
$q_{8}l_{4}$	8	4	96	80
$q_{8}l_{6}$	8	6	144	112
$q_{8}l_{8}$	8	8	192	144

**Table 7:** Experimental results: Accuracy computation after adversarial attack and purification using adversarial training (ϵ=0.3) for nine quantum circuit configurations with three qubit count $q_{4},q_{6},q_{8}$ and quantum layer count $l_{4},l_{6},l_{8}$—MNIST dataset.

ϵ Value	*q*	*l*	Clean Acc	FGSM		BIM		MIM		PGD	
				Attack	Pure	Attack	Pure	Attack	Pure	Attack	Pure
ϵ=0.3	4	4	97.80%	82.60%	84.70%	81.00%	84.80%	75.10%	79.90%	67.50%	70.90%
	4	6	97.86%	83.25%	84.65%	80.70%	83.28%	74.27%	80.78%	65.78%	69.63%
	4	8	96.91%	83.41%	84.04%	79.93%	83.05%	75.49%	79.65%	68.82%	71.87%
	6	4	98.50%	84.00%	86.10%	84.00%	86.00%	79.30%	82.60%	69.70%	72.60%
	6	6	98.87%	84.84%	86.91%	83.63%	84.15%	80.06%	81.49%	70.38%	71.14%
	6	8	98.91%	85.83%	86.95%	85.39%	86.99%	81.29%	82.83%	71.61%	72.72%
	8	4	98.02%	86.36%	87.72%	84.44%	85.82%	80.18%	81.79%	70.39%	73.64%
	8	6	97.92%	86.25%	88.53%	84.38%	85.64%	79.29%	80.63%	69.76%	72.88%
	8	8	97.86%	87.92%	89.88%	84.95%	85.90%	80.04%	81.84%	70.28%	71.91%
ϵ=0.5	4	4	98.00%	68.67%	70.47%	66.29%	69.38%	65.87%	67.28%	63.83%	66.21%
	4	6	97.72%	69.70%	71.80%	67.74%	69.83%	68.54%	69.28%	67.67%	68.25%
	4	8	97.69%	70.73%	72.94%	67.37%	69.84%	70.58%	71.38%	69.03%	70.47%
	6	4	98.52%	71.15%	73.49%	70.04%	71.64%	70.85%	71.55%	71.39%	72.19%
	6	6	97.77%	73.98%	75.74%	72.38%	73.37%	71.85%	73.36%	69.84%	71.97%
	6	8	97.69%	74.38%	76.85%	73.63%	75.31%	71.29%	73.73%	70.63%	72.49%
	8	4	96.98%	75.47%	77.73%	74.28%	76.60%	73.38%	74.94%	70.55%	73.32%
	8	6	97.74%	75.83%	78.29%	73.66%	75.82%	70.19%	73.28%	72.70%	73.07%
	8	8	98.06%	77.50%	79.10%	76.80%	78.10%	74.70%	75.48%	70.03%	73.74%

**Table 8:** Experimental results: Accuracy computation after adversarial attack and purification using adversarial training (ϵ=0.3) for nine quantum circuit configurations with three qubit count $q_{4},q_{6},q_{8}$ and quantum layer count $l_{4},l_{6},l_{8}$—Fashion MNIST dataset.

ϵ Value	*q*	*l*	Clean Acc	FGSM		BIM		MIM		PGD	
				Attack	Pure	Attack	Pure	Attack	Pure	Attack	Pure
ϵ=0.3	4	4	81.06%	49.47%	53.54%	46.38%	50.48%	40.64%	44.64%	39.38%	41.26%
	4	6	81.32%	48.62%	50.54%	45.17%	49.35%	39.74%	43.27%	38.29%	40.48%
	4	8	80.87%	48.74%	49.83%	45.06%	49.62%	40.49%	42.36%	39.37%	39.26%
	6	4	80.90%	50.80%	55.60%	48.00%	52.20%	43.10%	48.00%	42.07%	45.72%
	6	6	81.02%	51.11%	55.75%	48.67%	53.00%	43.88%	47.97%	42.56%	45.68%
	6	8	81.00%	52.04%	56.32%	48.99%	54.07%	44.62%	48.64%	41.85%	44.43%
	8	4	81.60%	53.15%	55.76%	49.65%	55.37%	43.71%	47.98%	41.93%	45.63%
	8	6	81.83%	53.22%	55.48%	50.52%	54.48%	44.49%	48.01%	42.91%	46.38%
	8	8	81.95%	54.92%	56.24%	51.51%	53.26%	45.05%	47.95%	43.84%	45.76%
ϵ=0.5	4	4	80.67%	43.70%	47.80%	42.50%	45.26%	33.50%	40.40%	26.70%	35.00%
	4	6	80.93%	43.45%	46.91%	41.67%	45.74%	34.65%	40.38%	27.43%	34.94%
	4	8	81.08%	44.36%	45.74%	40.24%	44.83%	35.77%	41.26%	28.67%	35.09%
	6	4	80.74%	45.85%	46.55%	41.74%	43.99%	35.56%	42.06%	29.52%	36.82%
	6	6	81.26%	46.92%	48.76%	41.13%	44.65%	35.87%	44.23%	30.88%	38.01%
	6	8	81.56%	46.67%	48.49%	43.56%	46.78%	36.38%	45.10%	32.36%	39.76%
	8	4	81.31%	47.41%	49.50%	43.85%	44.33%	36.64%	46.18%	34.57%	38.13%
	8	6	81.71%	48.69%	50.38%	44.92%	45.61%	37.59%	44.25%	35.81%	39.48%
	8	8	81.33%	50.53%	52.72%	41.84%	44.83%	38.60%	43.71%	35.52%	41.09%

**Table 9:** Attack Success Rate Comparison with Shi et al. [40]. Circuit configuration $q_{8}l_{8}$ is employed for comparison of outputs.

Attack (ϵ)	Shi et al. [40]				Proposed			
	MNIST		Fashion MNIST		MNIST		Fashion MNIST	
	Attack	Pure	Attack	Pure	Attack	Pure	Attack	Pure
FGSM (ϵ=0.1)	66.02%	97.66%	00.78%	76.56%	90.60%	93.16%	56.90%	58.70%
FGSM (ϵ=0.2)	08.59%	87.50%	00.39%	32.81%	85.20%	88.50%	53.94%	56.64%
BIM (ϵ=0.1)	21.09%	45.70%	0%	38.28%	88.30%	83.60%	55.33%	54.83%
BIM (ϵ=0.2)	21.09%	45.70%	0%	38.28%	80.20%	53.09%	51.82%	52.41%
MIM (ϵ=0.1)	19.14%	46.88%	0%	41.41%	83.43%	85.73%	50.02%	52.91%
MIM (ϵ=0.2)	19.14%	46.88%	0%	41.41%	79.69%	82.73%	47.23%	50.31%
PGD (ϵ=0.1)	25.00%	43.36%	0%	32.42%	77.89%	79.61%	39.31%	41.59%
PGD (ϵ=0.2)	03.12%	07.03%	0%	13.67%	73.37%	76.28%	35.62%	38.85%

**Table 10:** Comparison of classification accuracy for the MNIST dataset. The FGSM, iFGSM, and PGD attacks are compared with MagNet [33], DefenseGAN [34] and Collaborative defence GAN [36]. The classifier models employed for comparison in this table are adopted from Laykaviriyakul et al. [36]. The architecture of the three classifier models are shown in Figure 10.

Classifier Model	Attack	MagNet [33]	DefenseGAN [34]	Collaborative Defence GAN [36] Adv. Training: ϵ = 0.3	Proposed Adv. Training: ϵ = 0.3
Model 1	No Attack	98.42%	96.40%	95.70%	98.72%
	FGSM Attack (ϵ=0.3)	52.30%	76.12%	84.81%	89.88%
	iFGSM Attack (ϵ=0.3)	65.02%	85.71%	89.63%	85.90%
	PGD Attack (ϵ=0.3)	67.77%	84.40%	89.79%	73.94%
Model 2	No Attack	98.80%	96.80%	98.73%	98.84%
	FGSM Attack (ϵ=0.3)	94.60%	80.30%	49.65%	83.99%
	iFGSM Attack (ϵ=0.3)	96.31%	85.50%	40.27%	86.25%
	PGD Attack (ϵ=0.3)	96.16%	87.30%	71.06%	72.37%
Model 3	No Attack	89.32%	96.70%	98.65%	98.86%
	FGSM Attack (ϵ=0.3)	83.32%	78.06%	54.68%	85.64%
	iFGSM Attack (ϵ=0.3)	86.06%	89.08%	54.37%	83.68%
	PGD Attack (ϵ=0.3)	89.57%	88.44%	62.85%	75.01%

**Table 11:** Comparison of classification accuracy for the Fashion-MNIST dataset. The FGSM, iFGSM, and PGD attacks are compared with MagNet [33], DefenseGAN [34], and Collaborative defense GAN [36]. The models used for comparison in this table are adopted from Laykaviriyakul et al. [36]. The architecture of the three classifier models are shown in Figure 10.

Classifier Model	Attack	MagNet [33]	DefenseGAN [34]	Collaborative Defense GAN [36] Adv. Training: ϵ = 0.3	Proposed Adv. Training: ϵ = 0.3
Model 1	No Attack	88.34%	81.80%	89.95%	83.20%
	FGSM Attack (ϵ=0.3)	06.77%	49.40%	81.26%	57.04%
	iFGSM Attack (ϵ=0.3)	03.59%	71.43%	64.91%	70.18%
	PGD Attack (ϵ=0.3)	01.20%	70.24%	67.32%	69.04%
Model 2	No Attack	89.91%	83.74%	88.75%	85.06%
	FGSM Attack (ϵ=0.3)	89.29%	55.21%	01.14%	56.71%
	iFGSM Attack (ϵ=0.3)	83.84%	70.61%	00.03%	70.36%
	PGD Attack (ϵ=0.3)	85.64%	72.24%	00.69%	64.05%
Model 3	No Attack	89.60%	82.20%	88.78%	87.63%
	FGSM Attack (ϵ=0.3)	87.71%	55.92%	00.95%	59.03%
	iFGSM Attack (ϵ=0.3)	56.86%	75.92%	00.01%	71.14%
	PGD Attack (ϵ=0.3)	48.81%	74.80%	01.00%	68.41%

**Table 12:** Ablation study on classical generator model.

Dataset	ϵ-Value	Attack Type	Attack Accuracy	Pure Accuracy	Attack SSIM	Pure SSIM	Attack PSNR	Pure PSNR
MNIST	ϵ=0.3	FGSM	81.80%	83.10%	0.36	0.37	17.63	18.41
MNIST	ϵ=0.5	FGSM	74.40%	77.70%	0.34	0.35	15.52	16.83
FMNIST	ϵ=0.3	FGSM	51.37%	52.70%	0.34	0.36	15.01	16.12
FMNIST	ϵ=0.5	FGSM	43.18%	44.92%	0.33	0.34	14.62	15.34
MNIST	ϵ=0.3	BIM	80.40%	82.30%	0.35	0.36	16.63	17.41
MNIST	ϵ=0.5	BIM	73.71%	76.46%	0.33	0.34	14.16	15.26
FMNIST	ϵ=0.3	BIM	51.80%	54.70%	0.32	0.33	13.83	14.42
FMNIST	ϵ=0.5	BIM	41.56%	48.82%	0.32	0.33	14.03	14.92
MNIST	ϵ=0.3	MIM	76.60%	79.30%	0.34	0.36	16.26	17.02
MNIST	ϵ=0.5	MIM	43.10%	44.03%	0.33	0.34	16.17	16.94
FMNIST	ϵ=0.3	MIM	47.70%	50.90%	0.32	0.33	15.37	16.28
FMNIST	ϵ=0.5	MIM	37.70%	39.96%	0.31	0.33	15.04	16.84
MNIST	ϵ=0.3	PGD	67.50%	69.30%	0.33	0.34	15.73	16.98
MNIST	ϵ=0.5	PGD	50.10%	51.03%	0.33	0.34	15.73	16.98
FMNIST	ϵ=0.3	PGD	42.00%	43.30%	0.31	0.32	15.02	15.93
FMNIST	ϵ=0.5	PGD	34.38%	37.28%	0.30	0.31	14.94	15.27

**Table 13:** Ablation study on multiscaling discriminators—Accuracy after adversarial attack and purification in % using $q_{8}l_{8}$ quantum circuit where ✔ indicates inclusion and **✗** indicates exclusion.

Dataset (ϵ-Value)	D_1_ 28×28	D_2_ 14×14	D_3_ 7×7	FGSM Attack Acc	FGSM Pure Acc	BIM Attack Acc	BIM Pure Acc	MIM Attack Acc	MIM Pure Acc	PGD Attack Acc	PGD Pure Acc
MNIST (ϵ=0.3)	✔	✔	✔	87.92	89.88	84.95	85.90	80.04	81.84	70.28	71.91
	✔	✔	**✗**	85.88	87.33	82.51	83.11	78.48	78.63	68.26	69.61
	✔	**✗**	**✗**	84.72	86.55	80.97	81.52	77.15	76.63	66.92	68.02
MNIST (ϵ=0.5)	✔	✔	✔	77.50	79.10	76.80	78.10	74.70	75.48	70.03	73.74
	✔	✔	**✗**	75.83	77.49	74.14	77.18	73.93	73.41	68.97	71.86
	✔	**✗**	**✗**	74.91	76.76	72.86	75.43	66.52	71.11	66.73	69.28
FMNIST (ϵ=0.3)	✔	✔	✔	54.92	56.24	51.51	53.26	45.05	47.95	43.84	45.76
	✔	✔	**✗**	52.78	54.75	50.02	51.30	43.74	45.82	41.24	43.63
	✔	**✗**	**✗**	51.39	52.53	48.49	49.81	41.00	43.63	40.18	41.82
FMNIST (ϵ=0.5)	✔	✔	✔	50.53	52.72	41.84	44.83	38.60	43.71	35.52	41.09
	✔	✔	**✗**	48.73	50.18	39.81	42.06	37.04	41.93	33.11	40.27
	✔	**✗**	**✗**	47.75	48.20	37.72	40.69	35.94	40.03	31.47	38.29

**Table 14:** Ablation study on multiscaling discriminators—SSIM and PSNR values after adversarial attack where ✔ indicates inclusion and **✗** indicates exclusion.

Dataset (ϵ-Value)	D_1_ 28×28	D_2_ 14×14	D_3_ 7×7	FGSM SSIM	FGSM PSNR	BIM SSIM	BIM PSNR	MIM SSIM	MIM PSNR	PGD SSIM	PGD PSNR
MNIST (ϵ=0.3)	✔	✔	✔	0.37	21.41	0.35	20.72	0.33	20.12	0.31	19.72
	✔	✔	**✗**	0.35	19.12	0.34	19.07	0.30	18.43	0.29	17.23
	✔	**✗**	**✗**	0.32	19.03	0.27	19.03	0.29	17.02	0.30	16.02
MNIST (ϵ=0.5)	✔	✔	✔	0.35	19.31	0.34	19.21	0.33	17.92	0.30	16.55
	✔	✔	**✗**	0.33	18.83	0.32	18.74	0.30	17.44	0.27	16.06
	✔	**✗**	**✗**	0.32	18.14	0.30	17.26	0.27	14.01	0.26	15.45
FMNIST (ϵ=0.3)	✔	✔	✔	0.30	19.41	0.28	18.74	0.27	17.67	0.25	17.29
	✔	✔	**✗**	0.28	18.20	0.26	17.01	0.26	16.02	0.24	16.18
	✔	**✗**	**✗**	0.26	17.17	0.25	16.82	0.25	15.34	0.23	15.90
FMNIST (ϵ=0.5)	✔	✔	✔	0.28	18.05	0.27	17.53	0.25	16.00	0.23	16.16
	✔	✔	**✗**	0.25	16.26	0.25	16.82	0.23	15.08	0.22	14.17
	✔	**✗**	**✗**	0.23	16.64	0.24	16.04	0.23	14.34	0.21	14.66

**Table 15:** Ablation study on Loss by varying the different types of loss, where ✔ indicates inclusion and **✗** indicates exclusion.

$L_{\mathrm{Sal}}$	$L_{\mathrm{Class}}$	$L_{\mathrm{Rec}}$	$L_{\mathrm{Adv}}$	$L_{\mathrm{Fea}}$	Accuracy
**✗**	✔	✔	✔	✔	80.80
✔	**✗**	✔	✔	✔	77.60
✔	✔	**✗**	✔	✔	85.30
✔	✔	✔	**✗**	✔	84.80
✔	✔	✔	✔	**✗**	83.00

## Data Availability

The raw data supporting the conclusions of this article will be made available by the authors on request.

## Abbreviations

The following abbreviations are used in this manuscript:

AI	Artificial Intelligence
AML	Adversarial Machine Learning
AWS	Amazon Web Services
BIM	Basic Iterative Method
CNOT	Controlled-NOT
CNN	Convolutional Neural Network
CW	Carlini & Wagner
FGSM	Fast Gradient Sign Method
F-MNIST	Fashion Modified National Institute of Standards and Technology
GAN	Generative Adversarial Network
GenAI	Generative AI
HQCGAN	Hybrid quantum-classical GAN
HQMSGAN	Hybrid Quantum Multi-Scale Generative Adversarial Network
iFGSM	iterative FGSM
MIM	Momentum Iterative Method
ML	Machine Learning
MNIST	Modified National Institute of Standards and Technology
NISQ	Noisy Intermediate Scale Quantum
PGD	Projected Gradient Descent
PSNR	Peak Signal-to-Noise Ratio
PQC	Parameterized Quantum Circuit
QAML	Quantum Adversarial Machine Learning
QCGAN	Quantum Classical Conditional GAN
QCNN	Quantum Convolutional Neural Network
QGAN	Quantum Generative Adversarial Network
QRBM	Quantum Restricted Boltzmann Machine
QML	Quantum Machine Learning
SSIM	Structural Similarity Index Measure
SV1	State Vector 1
TN1	Tensor Network 1
VQC	Variational Quantum Circuit
WGAN	Wasserstein GAN
WGAN-GP	Wasserstein GAN-Gradient penalty
